# Stereoselective Luche Reduction of Deoxynivalenol and Three of Its Acetylated Derivatives at C8

**DOI:** 10.3390/toxins6010325

**Published:** 2014-01-10

**Authors:** Philipp Fruhmann, Christian Hametner, Hannes Mikula, Gerhard Adam, Rudolf Krska, Johannes Fröhlich

**Affiliations:** 1 Institute of Applied Synthetic Chemistry, Vienna University of Technology, Getreidemarkt 9, Vienna 1060, Austria; E-Mails: christian.hametner@tuwien.ac.at (C.H.); hannes.mikula@tuwien.ac.at (H.M.); johannes.froehlich@tuwien.ac.at (J.F.); 2 Department of Applied Genetics and Cell Biology, University of Natural Resources and Life Sciences, Vienna (BOKU), Konrad Lorenz Str. 24, Tulln 3430, Austria; E-Mail: gerhard.adam@boku.ac.at; 3 Department for Agrobiotechnology (IFA-Tulln), Center for Analytical Chemistry, University of Natural Resources and Life Sciences, Vienna (BOKU), Konrad Lorenz Str. 20, Tulln 3430, Austria; E-Mail: rudolf.krska@boku.ac.at

**Keywords:** dihydroxycalonectrins, trichothecenes, DON, Luche reduction, scirpene

## Abstract

The trichothecene mycotoxin deoxynivalenol (DON) is a well known and common contaminant in food and feed. Acetylated derivatives and other biosynthetic precursors can occur together with the main toxin. A key biosynthetic step towards DON involves an oxidation of the 8-OH group of 7,8-dihydroxycalonectrin. Since analytical standards for the intermediates are not available and these intermediates are therefore rarely studied, we aimed for a synthetic method to invert this reaction, making a series of calonectrin-derived precursors accessible. We did this by developing an efficient protocol for stereoselective Luche reduction at C8. This method was used to access 3,7,8,15-tetrahydroxyscirpene, 3-deacetyl-7,8-dihydroxycalonectrin, 15-deacetyl-7,8-dihydroxycalonectrin and 7,8-dihydroxycalonectrin, which were characterized using several NMR techniques. Beside the development of a method which could basically be used for all type B trichothecenes, we opened a synthetic route towards different acetylated calonectrins.

## 1. Introduction

Trichothecene based mycotoxins are common and widespread contaminants in food and feed. They can affect human and animal health by causing several acute and chronic symptoms after uptake [1]. Toxicity studies showed that the primary mode of action of trichothecenes is inhibition of eukaryotic protein synthesis [2,3,4]. When consumed in contaminated foods, trichothecenes can act as neurotoxin, immunosuppressive or nephrotoxin [5,6,7].

**Scheme 1:**
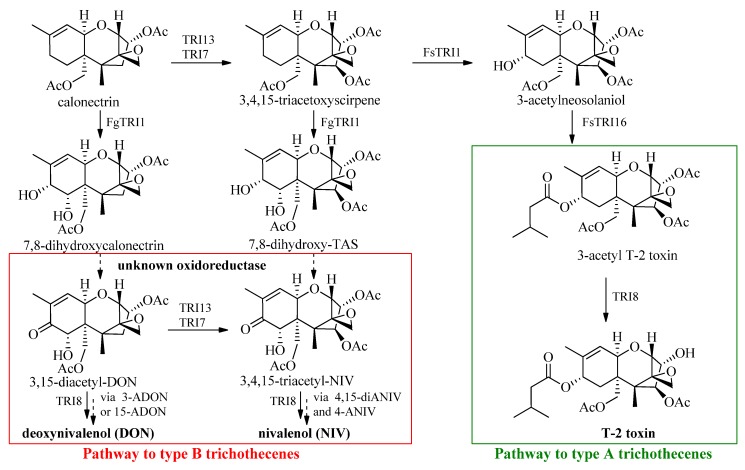
.Biosynthetic pathway of Type A and Type B trichothecenes (modified from [10]). *F. graminearum* (Fg) and *F. sporotrichioides* (Fs) gene products catalyzing the reactions are indicated.

The polarity of trichothecenes depends on the number of hydroxyl groups (ranging from 1 to 5) and their esterification status. So far, more than 200 different trichothecenes have been reported, which are produced by different genera such as *Fusarium*, *Myrothecium*, *Stachybotrys*, *Cephalosporium*, *Trichoderma* and *Trichothecium* [8]. Generally, they are divided into four different groups (A–D), all containing a tricyclic 12,13-epoxytrichothec-9-ene core structure [9]. Type A toxins are compounds with at least one hydroxyl group, either no oxygen substituent at C8 or an ester functionality. In contrast, type B trichothecenes feature a carbonyl functionality at C8. The most prominent toxins of the two classes mentioned above are T-2 toxin (type A), nivalenol (NIV, type B) and deoxynivalenol (DON, type B). From the biosynthetic point of view type A and type B trichothecenes are derived from the same precursors (Scheme 1) and most of the responsible genes are already described in the literature [10].

The oxidoreductase step leading to the C8 keto group is still uncharacterized. Our recent findings [11] showed the occurrence of pentahydroxyscirpene (PHS), a NIV derivative with an OH function at C8 which was isolated in substantial amounts (10%–20%) together with NIV after fermentation and also in artificially inoculated wheat. Other results showed the occurrence of 7,8-dihydroxycalonectrin [12,13,14,15] alone or in combination with 15-deacetyl-7,8-dihydroxycalonectrin [16] or 3,7,8,15-tetrahydroxyscirpene [17]. Since these compounds are all supposed to be toxin precursors, the findings suggest that there are even more acetylated forms and derivatives of trichothecene precursors that might also be present in contaminated grain, but which are not studied due to lack of standards. Therefore, we have focused on developing a reliable method to make this substance class accessible.

## 2. Synthetic Approach

### 2.1. General Aspects

**Scheme 2:**
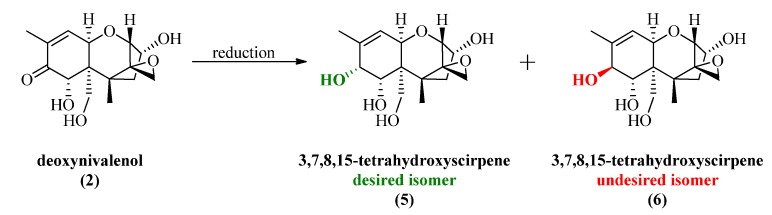
Desired and undesired isomer of 3,7,8,15-tetrahydroxyscirpene via reduction of deoxynivalenol (DON).

The most obvious synthetic way to access 7,8-dihydroxycalonectrin derivatives and other trichothecenes with a C8 hydroxy group is the selective reduction of the C8 carbonyl function. One common characteristic of naturally occurring compounds like trichothecenes is a very well defined stereochemistry with a lot of chiral information. For example, DON has seven stereogenic centers, which influences the synthetic introduction of a new stereocenter in a very unpredictable way. Introducing a new hydroxyl group in position 8 would therefore lead to a mixture of 3,7,8,15-tetrahydroxyscirpene with its undesired isomer (Scheme 2).

To avoid formation of the undesired isomer and suppress side reactions of the hydride reagent, we choose to utilize the Luche reduction to achieve a very selective method for the reduction of DON.

### 2.2. Luche Reduction

**Scheme 3:**
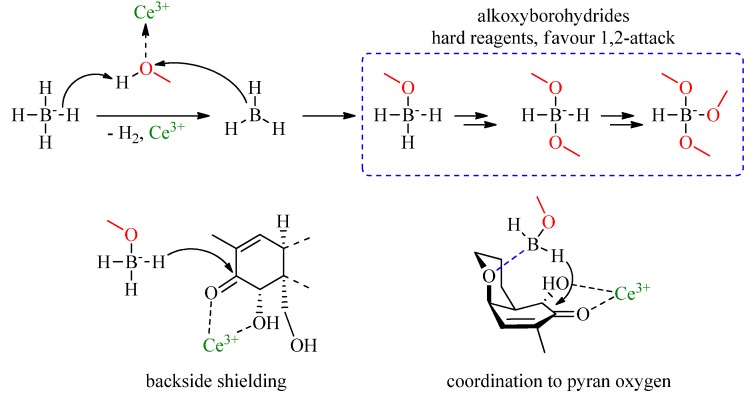
Mechanism of alkoxyborohydride formation, shielding and coordination.

The Luche reduction [18,19,20] can be used to convert α, β-unsaturated ketones into allylic alcohols using CeCl_3_, NaBH_4_ and methanol as solvent. The main role of cerium(III) chloride is to coordinate with the alcohol solvent, making its proton more acidic which can then be abstracted by the carbonyl oxygen of the ketone. After addition of NaBH_4_ it also reacts with the cerium activated alcohol forming a series of alkoxyborohydrides (Scheme 3). Since alkoxyborohydrides are “hard reagents” their formation results in a selective 1,2-hydride attack on the protonated carbonyl group which leads to the desired reaction. In addition the use of CeCl_3_ offers the possibility of coordinating [21] with the C7 hydroxy group, which results in a shielding of the backside of deoxynivalenol (Scheme 3). Due to this shielding effect, the desired frontside hydride attack should be more favored. The last point which might have an influence on the reaction, is the oxygen in the pyran ring of DON. Since this oxygen is located next to the reaction site, it is possible, that a coordination between the activated borohydride species and the oxygen is taking place (Scheme 3), which would lead to an even more targeted reduction.

## 3. Results and Discussion

### 3.1. Method Development

**Scheme 4:**
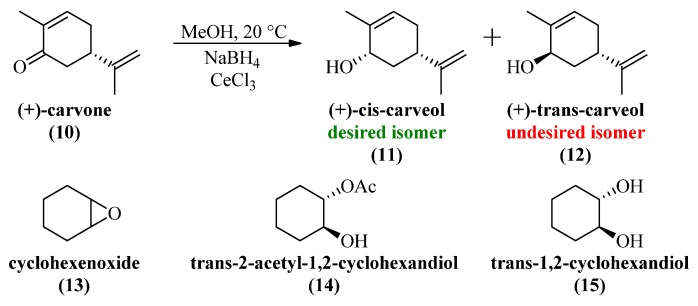
(+)-Carvone and its possible reaction products as model for deoxynivalenol.

Since DON and its acetylated derivatives are very expensive we decided to use (+)-carvone (Scheme 4) as a cheap and readily available mimic for method evaluation. Although the steric information is quite simple compared with deoxynivalenol, it provides a good model for method optimization. Therefore, the lowest possible concentration of all involved reagents as well as the estimation of possible side reactions was examined, in order to avoid needless loss of starting material. In addition **13** was used as stability test for the epoxy group, **14** as stability estimation for acetyl groups and **15** as mimic for the coordination effect in deoxynivalenol.

For these experiments we used CeCl_3_·7H_2_O as it is significantly better soluble in methanol than the anhydrous form. After some tests with varying equivalents, the reactions with 1 equivalent NaBH_4_ and 0.5 equivalents CeCl_3_·7H_2_O for 1 equivalent carvone turned out to be superior to other ratios, allowing full stereoselective conversion without too much decomposition. With these conditions we were able to perform the reaction from carvone to (+)-*cis*-carveol in 30 min with an isolated yield of 92%. All attempts with lower reagent concentrations ended up with bad conversions (7%–13% of starting material left after 24 h), more byproducts (>10%) and elongated reaction times (>24 h). Stability testing regarding the epoxy group as well as the stability of the acetyl mimic under reaction conditions revealed slow decomposition over time which rose to 16% within 2 h.

### 3.2. Synthesis of 3-ADON, 15-ADON, 3,15-diADON and Their Reduction

**Scheme 5:**
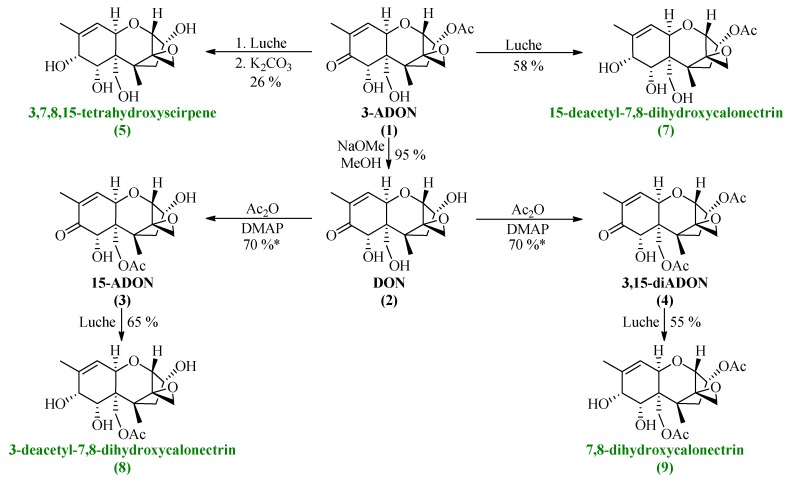
Synthetic approach towards the different DON derivatives including reduction under Luche conditions (NaBH_4_, CeCl_3_) to the corresponding calonectrins. (* = yield as sum of both products).

3-ADON was obtained from BOKU, Dept. for Agrarbiotechnology (IFA-Tulln) and was spectroscopically pure (NMR, Figure S1) in accordance with existing literature [22]. The synthetic route towards the different acetylated DON derivatives was carried out by deprotection of 3-ADON via NaOMe/MeOH followed by Steglich esterification [23] to obtain 15-ADON and 3,15-diADON simultaneously (Scheme 5).

### All synthesized DON derivatives were reduced applying an optimized Luche protocol (See *3.3. Reduction Protocol*) leading to the desired 7,8-dihydroxycalonectrin derivatives in moderate to good yields.

### 3.3. Reduction Protocol

All Luche reductions towards the different derivatives were done with 0.05–0.30 mmol of DON or its corresponding acetylated form as starting material. The general procedure therefore was: Toxin (1.00 equ.) was dissolved in 1 mL MeOH and CeCl_3_·7H_2_O (0.50 equ. in 1 mL MeOH) was added. NaBH_4_ (1.00 equ.) was dissolved in 1 mL MeOH and added with moderate speed (dropwise, but fast to prevent rising pressure due to H_2_ formation). We recommend preparation of a stock solution of CeCl_3_·7H_2_O and NaBH_4_ as it is easier to deal with the low substance amounts. In case of NaBH_4_ the solution should be prepared just in time and used quickly to avoid evolving hydrogen. After addition of all reagents, the reaction was stirred at room temperature until TLC revealed conversion of the starting material. The mixture was concentrated without heating under reduced pressure to avoid decomposition and directly subjected to column chromatography.

### 3.4. Spectroscopic Investigation

**Figure 1:**
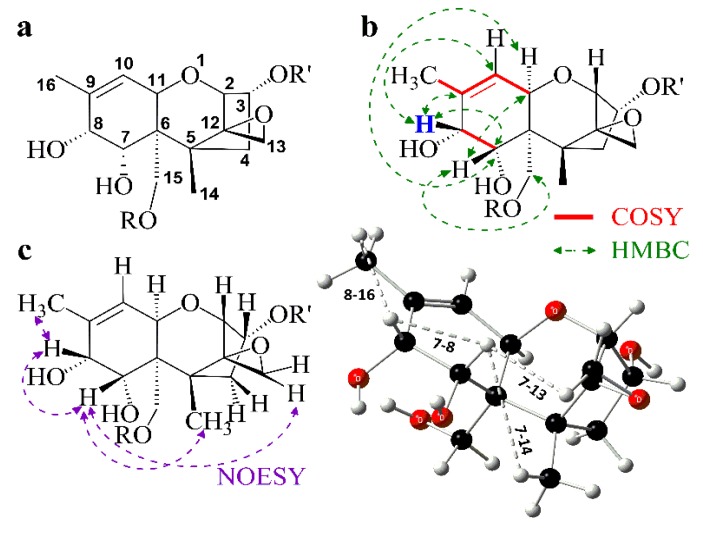
(**a**) Systematic numbering of trichothecenes; (**b**) Selected COSY and HMBC correlations of **5** (R = R' = H), **7** (R = H, R' = Ac) and **8** (R = Ac, R' = H); (**c**) Selected NOESY correlations within the 2D (left) and 3D (right, optimized geometry, Figure S30, Tables S1,2) structure of **5**.

To ensure that the desired stereochemistry of the product was achieved as well as for a full characterization including proof of stereochemistry, all products were investigated via several NMR techniques. In case of 7,8-dihydroxycalonectrin which is already well characterized in the literature, we obtained the identical spectroscopic information as published [22] by recording ^1^H and ^13^C spectra. In the case of the other three products we also recorded COSY, HSQC, HMBC and NOESY spectra to achieve a complete characterization (Figure 1, Table 1).

**Table 1:** ^1^H NMR data (in methanol-*d_4_*) including chemical shifts, (multiplicity) and [coupling constants] of the isolated products. Multiplicities are abbreviated as s (singlet), d (doublet), t (triplet), q (quartet), m (multiplet), and b (broad signal). R' and R are referring to the methyl signal of the attached acetyl substituent in position 3 (R') and 15 (R).

Product	2 (d)	3 (dt)	4α (dd)	4β (dd)	7β (d)	8β (d)	10 (dq)	11 (d)
(**5**)	3.38 [4.5]	4.31 [10.7, 4.5]	2.21 [14.6, 4.5]	1.97 [14.6, 10.7]	4.43 [4.7]	3.91 [4.7]	5.57 [5.6] (b)	4.40 [5.6]
(**7**)	3.68 [4.4]	5.05 [11.1, 4.4]	2.48 [15.0, 4.4]	2.04 [15.0, 11.1]	4.40 [5.0]	3.91 [5.0]	5.54 [5.5, 1.4]	4.33 [5.5]
(**8**)	3.41 [4.4]	4.30 [11.1, 4.4]	2.49 [14.6, 4.4]	1.97 [14.6, 11.1]	4.45 [5.6]	3.92 [5.6]	5.57 [5.8, 1.4]	4.64 [5.8]
(**9**)	3.72 [4.4]	5.05 [11.2, 4.4]	2.68 [15.1, 4,4]	1.95–2.15(m)	4.42 [5.2]	3.91 [5.2]	5.53 [5.9, 1.5]	4.57 [5.9]
**Product**	**13a (d)**	**13b (d)**	**14 (s)**	**15a (d)**	**15b (d)**	**16 (s)**	**3 R' (s)**	**15 R (s)**
(**5**)	3.02 [4.4]	3.16 [4.4]	1.14	3.64 [12.6]	3.88 [12.6]	1.84	---	---
(**7**)	3.08 [4.1]	3.20 [4.1]	1.17	3.67 [12.6]	3.90 [12.6]	1.84	---	2.10
(**8**)	3.04 [4.4]	3.18 [4.4]	1.15	4.34 [12.6]	4.38 [12.6]	1.85	2.04	---
(**9**)	3.09 [4.3]	3.22 [4.3]	1.18	4.37(s), 2H		1.85	2.04	2.10

## 4. Experimental Section

### 4.1. General

Thin layer chromatography (TLC) was performed over silica gel 60 F254 (Merck). All chromatograms were visualized by heat staining using ceric ammonium molybdate/Hanessian’s stain [24] in ethanol/sulfuric acid. Chromatographic separation was done on silica gel 60 (40–63 µm, Merck, Darmstadt, Germany) using a SepacoreTM Flash System (Büchi, Switzerland). ^1^H and ^13^C NMR spectra were recorded on an Avance DRX-400 MHz spectrometer as well as at a Bruker DPX-200 spectrometer (Bruker, Karlsruhe, Germany). Data were recorded and evaluated using TOPSPIN 1.3 (Bruker Biospin, Karlsruhe, Germany). CD spectra were recorded using a JASCO J-815 CD spectrometer (JASCO, Easton, MD, USA), and can be found in the supporting information. All chemical shifts are given in ppm relative to tetramethylsilane. The calibration was done using residual solvent signals [25]. Multiplicities are abbreviated as s (singlet), d (doublet), t (triplet), q (quartet) and b (broad signal). 3-ADON was obtained from University of Natural Resources and Life Sciences, Vienna, Dept. for Agrobiotechnology (IFA-Tulln) and was used after ^1^H NMR purity check [22]. All other chemicals were purchased from Sigma-Aldrich (Schnelldorf, Germany).

### 4.2. Deoxynivalenol (**2**)

3-ADON (85.6 mg, 0.25 mmol) was dissolved in 5 mL dry methanol and NaOMe (13.7 mg, 0.25 mmol) was added to the reaction. After 1.5 h TLC revealed full conversion of the starting material and the reaction mixture was concentrated to 1 mL. Finally the solution was directly purified by the use of column chromatography (CHCl_3_:MeOH = 9:1) which yielded deoxynivalenol (79.0 mg, 95%) as white solid. The reaction product was proved to be identical to an authentic sample by TLC and thus was used for the next step.

### 4.3. 15-ADON (3) and 3,15-diADON (**4**)

DON (79.0 mg, 0.27 mmol) was dissolved in 50 mL dry dichloromethane. Pyridine (1 mL) and 4-DMAP (app. 10 mg) were added followed by the dropwise addition of acetic anhydride (27.2 mg, 0.27 mmol). The reaction was stirred overnight, treated with 20 mL HCl (2 N) and extracted 3 times with 50 mL dichloromethane. After drying with Na_2_SO_4_, filtration and evaporation of the solvent the remaining residue was subjected to column chromatography (CHCl_3_:MeOH = 95:5) to yield 15-ADON (42.0 mg, 47%, Figures S2,S3) and 3,15-diADON (23.5 mg, 23%, Figures S4,S5) as white solid. Total yield = 70%, 93% conversion. 15-ADON (3): ^1^H NMR (200 MHz, CDCl_3_) δ = 6.61 (dq, *J* = 5.7, 1.6 Hz, 1H), 4.89 (d, *J* = 5.7 Hz, 1H), 4.83 (d, *J* = 1.6 Hz, 1H), 4.52 (dt, *J* = 10.2, 4.7 Hz, 1H), 4.24 (s, 2H), 3.78 (d, *J* = 1.8 Hz, 1H), 3.63 (d, *J* = 4.5 Hz, 1H), 3.13 (d, *J* = 4.3 Hz, 1H), 3.08 (d, *J* = 4.3 Hz, 1H), 2.22 (dd, *J* = 14.8, 4.7 Hz, 1H), 2.08 (dd, *J* = 14.7, 10.4 Hz, 1H), 1.88 (s, 3 H), 1.87 (s, 3H), 1.07 (s, 3H); ^13^C-NMR (50 MHz, CDCl_3_) δ = 199.6 (s), 170.3 (s), 138.8 (d), 135.6 (s), 80.7 (d), 73.5 (d), 70.1 (d), 68.9 (s), 65.5 (s), 62.2 (t), 51.4 (s), 47.4 (t), 46.3 (s), 43.3 (t), 20.7 (q), 15.4 (q), 13.8 (q). **3,15-diADON (4)**: ^1^H NMR (200 MHz, CDCl_3_) δ = 6.56 (dq, *J* = 5.8, 1.4 Hz, 1H), 5.20 (dt, *J* = 10.9, 4.6 Hz, 1H), 4.80 (d, *J* = 2.0 Hz, 1H), 4.69 (d, *J* = 5.8 Hz, 1H), 4.27 (d, *J* = 12.1 Hz, 1H), 4.20 (d, *J* = 12.1 Hz, 1H), 3.89 (d, *J* = 4.3 Hz, 1H), 3.80 (d, *J* = 2.0 Hz, 1H), 3.14 (d, *J* = 4.3 Hz, 1H), 3.09 (d, *J* = 4.3 Hz, 1H), 2.31 (dd, *J* = 15.2, 4.8 Hz, 1H), 2.15 (dd, J = 15.2, 10.9 Hz, 1H), 2.12 (s, 3H), 1.88 (s, 3 H), 1.87 (s, 3H), 1.08 (s, 3H); ^13^C-NMR (50 MHz, CDCl_3_) δ = 199.3 (s), 170.3 (s), 170.2 (s), 138.4 (d), 135.6 (s), 78.9 (d), 73.4 (d), 71.1 (d), 70.1 (d), 64.9 (s), 62.1 (t), 51.5 (s), 47.4 (t), 45.8 (s), 40.3 (t), 21.0 (q), 20.6 (q), 15.3 (q), 13.6 (q); ^1^H NMR data consistent with the ones reported in literature [26,27].

### 4.4. (+)-cis-Carveol (**11**) (Large Scale Luche Reduction)

(+)-Carvone (10) (3.00 g, 20.0 mmol) and CeCl_3_·7H_2_O (1.86 g, 5.0 mmol) were dissolved in 150 mL MeOH and cooled to 0 °C. NaBH_4_ (0.76 g, 20.0 mmol) was dissolved in 100 mL MeOH and added within 5 mins to the reaction solution via a dropping funnel. After complete addition of the NaBH_4_-solution the cooling bath was removed and the reaction continued. TLC control (hexane:EtOAc = 5:1) after 30 min revealed complete conversion of the starting material and the reaction was treated with 50 mL 2N HCl and extracted three times with 100 mL Et_2_O. The organic phase was dried over Na_2_SO_4_, filtered and the solvent was removed under reduced pressure. Column chromatography (hexane:EtOAc = 5:1) yielded 2.79 g (92%, Figures S6,S7) of a slightly yellow oil which was consistent with literature NMR data [28,29] for (+)-*cis*-carveol. ^1^H NMR (200 MHz, CDCl_3_) δ = 5.35 (b, 1H), 4.62 (b, 1H), 4.08 (b, 1H), 3.77 (b, 1H), 1.50 – 2.30 (m, 11H), 1.40 (dt, *J* = 12.2, 10.0 Hz, 1H); ^13^C-NMR (50 MHz, CDCl_3_) δ = 148.7 (s, C=), 136.6 (s, C=), 123.3 (d, =CH), 108.8 (t, =CH_2_), 70.3 (d, CH), 40.7 (d, CH), 37.8 (t, CH_2_), 31.0 (t, CH_2_), 20.3 (q, CH_3_), 19.0 (q, CH_3_).

### 4.5. Conversion of 3-ADON (**1**) to 15-Deacetyl-7,8-dihydroxycalonectrin (**7**) and 3,7,8,15-Tetrahydroxyscirpene (**5**)

3-ADON (1.00 equ.) was dissolved in 1 mL MeOH and CeCl_3_·7H_2_O (0.50 equ. in 1 mL MeOH) was added. NaBH_4_ (1.00 equ.) was dissolved in 1 mL MeOH and added with moderate speed (dropwise, but fast enough to prevent rising pressure due to H_2_ formation). After completion the reaction was stirred until TLC revealed conversion of the starting material. The mixture was concentrated without heating under reduced pressure and subjected directly to column chromatography (DCM:MeOH = 9:1) to yield 10.6 mg (58%, Figures S13–S17) of 15-deacetyl-7,8-dihydroxycalonectrin. In order to obtain 3,7,8,15-tetrahydroxyscirpene (5), the whole reaction was repeated and evaporated to dryness. After uptake in 3 mL dry MeOH, K_2_CO_3_ (2.00 equ.) was added and the reaction stirred until TLC indicated the deprotection of the acetyl group in position 3. The reaction mixture was concentrated to 1 mL and purified via column chromatography (CHCl_3_:MeOH = 9:1) to yield 13.6 mg (26% for two steps, Figures S8-S12) of 3,7,8,15-tetrahydroxyscirpene. 3,7,8,15-Tetrahydroxyscirpene (5): ^1^H NMR (400 MHz, methanol-*d_4_*) δ = 5.57 (bd, *J* = 5.6 Hz, 1H), 4.43 (d, *J* = 4.7 Hz, 1H), 4.40 (d, *J* = 5.6 Hz, 1H), 4.31 (dt, *J* = 10.7, 4.5 Hz, 1H), 3.91 (d, *J* = 4.7 Hz, 1H), 3.88 (d, *J* = 12.6 Hz, 1H), 3.64 (d, *J* = 12.6 Hz, 1H), 3.38 (d, *J* = 4.5 Hz, 1H), 3.16 (d, *J* = 4.4 Hz, 1H), 3.02 (d, *J* = 4.4 Hz, 1H), 2.21 (dd, *J* = 14.6, 4.5 Hz, 1H), 1.97 (dd, *J* = 14.6, 10.7 Hz, 1H), 1.84 (s, 3 H), 1.14 (s, 3H); ^13^C-NMR (100 MHz, methanol-*d_4_*) δ = 140.0 (s, C-9), 124.3 (d, C-10), 81.1 (d, C-2), 72.9 (d, C-11), 72.2 (d, C-8), 72.0 (d, C-7), 69.8 (d, C-3), 66.5 (s, C-12), 62.3 (t, C-15), 48.6 (s, C-6), 48.5 (s, C-13), 47.7 (s, C-5), 45.8 (t, C-4), 20.8 (q, C-16), 16.2 (q, C-14). The signal at 48.5 (C-13) is not visible in the ^13^C spectra, but could be located in the correlated spectra. HRMS (APCI^+^): m/z calcd for (5) [M + Na^+^]: 321.1309; found: 321.1305. **15-Deacetyl-7,8-dihydroxycalonectrin** (7): ^1^H NMR (400 MHz, methanol-*d_4_*) δ = 5.54 (dq, *J* = 5.5, 1.4 Hz, 1H), 5.05 (dt, *J* = 11.1, 4.4 Hz, 1H), 4.40 (d, *J* = 5.0 Hz, 1H), 4.33 (d, *J* = 5.5 Hz, 1H), 3.91 (d, *J* = 5.0 Hz, 1H), 3.90 (d, *J* = 12.6 Hz, 1H), 3.68 (d, *J* = 4.4 Hz, 1H), 3.67 (d, *J* = 12.6 Hz, 1H), 3.20 (d, *J* = 4.1 Hz, 1H), 3.08 (d, *J* = 4.1 Hz, 1H), 2.48 (dd, *J* = 15.0, 4.4 Hz, 1H), 2.10 (s, 3H), 2.04 (dd, *J* = 15.0, 11.1 Hz, 1H), 1.84 (s, 3 H), 1.17 (s, 3H); ^13^C-NMR (100 MHz, methanol-*d_4_*) δ = 172.6 (s, acetyl C=O), 140.4 (s, C-9), 123.7 (d, C-10), 79.9 (d, C-2), 73.0 (d, C-3), 72.9 (d, C-11), 72.1 (d, C-8), 71.7 (d, C-7), 66.1 (s, C-12), 62.1 (t, C-15), 48.7 (t, C-13), 48.5 (s, C-6), 47.1 (s, C-5), 42.6 (t, C-4), 20.9 (q, acetyl CH_3_), 20.8 (q, C-16), 15.9 (q, C-14). HRMS (APCI^+^): m/z calcd for (7) [M + Na^+^]: 363.1414; found: 363.1409.

### 4.6. Reduction of 15-ADON (**3**) and 3,15-diADON (**4**) to 3-Deacetyl-7,8-dihydroxycalonectrin (*8*) and 7,8-Dihydroxycalonectrin (**9**)

Toxin (1.00 equ.) was dissolved in 1 mL MeOH and CeCl_3_·7H_2_O (0.50 equ. in 1 mL MeOH) was added. NaBH_4_ (1.00 equ.) was dissolved in 1 mL MeOH and added with moderate speed (dropwise, but fast to prevent rising pressure due to H_2_ formation). After completion the reaction was stirred until TLC revealed conversion of the starting material. The mixture was concentrated without heating under reduced pressure and directly subjected to column chromatography (DCM:MeOH = 97.5:2.5 for 7,8-dihydroxycalonectrin and DCM:MeOH = 9:1 for 3-deacetyl-7,8-dihydroxycalonectrin) to yield the desired products in 55% (7,8-dihydroxycalonectrin, Figures S23,S24) and 65% (3-deacetyl-7,8-dihydroxycalonectrin, Figures S18–S22). **7,8-Dihydroxycalonectrin (9)**: ^1^H NMR (200 MHz, methanol-*d_4_*) δ = 5.53 (dq, *J* = 5.9, 1.5 Hz, 1H), 5.05 (dt, *J* = 11.2, 4.4 Hz, 1H), 4.52 (d, *J* = 5.9 Hz, 1H), 4.42 (d, *J* = 5.3 Hz, 1H), 4.40 (s, 2H), 3.91 (d, *J* = 5.3 Hz, 1H), 3.72 (d, *J* = 4.4 Hz, 1H), 3.22 (d, *J* = 4.3 Hz, 1H), 3.09 (d, *J* = 4.2 Hz, 1H), 2.68 (dd, *J* = 15.1, 4.1 Hz, 1H), 1.95-2.15 (m, 1H), 2.10 (s, 3H), 2.04 (s, 3H), 1.85 (s, 3 H), 1.18 (s, 3H); ^13^C-NMR (50 MHz, methanol-*d_4_*) δ = 172.6 (s), 172.4 (s), 141.9 (s), 122.1 (d), 80.1 (d), 72.9 (d), 72.3 (d), 71.4 (d), 71.3 (d), 66.3 (s), 65.4 (t), 47.8 (s), 46.8 (s), 43.0 (t), 21.2 (q), 20.9 (q), 20.8 (q), 15.8 (q, C-14). 1 Signal missing due to solvent overlap. HRMS (APCI^+^): m/z calcd for (**9**) [M+Na^+^]: 405.1520; found: 405.1515. **3-Deacetyl-7,8-dihydroxycalonectrin (8)**: ^1^H NMR (400 MHz, methanol-*d_4_*) δ = 5.57 (dq, *J* = 5.8, 1.4 Hz, 1H), 4.64 (d, *J* = 5.8 Hz, 1H), 4.45 (d, *J* = 5.6 Hz, 1H), 4.38 (d, *J* = 12.6 Hz, 1H), 4.34 (d, *J* = 12.6 Hz, 1H), 4.30 (dt, *J* = 11.1, 4.4 Hz, 1H), 3.92 (d, *J* = 5.6 Hz, 1H), 3.41 (d, *J* = 4.4 Hz, 1H), 3.18 (d, *J* = 4.4 Hz, 1H), 3.04 (d, *J* = 4.4 Hz, 1H), 2.49 (dd, *J* = 14.6, 4.4 Hz, 1H), 2.04 (s, 3H), 1.97 (dd, *J* = 14.6, 11.1 Hz, 1H), 1.85 (s, 3 H), 1.15 (s, 3H); ^13^C-NMR (100 MHz, methanol-*d_4_*) δ = 172.5 (s, acetyl C=O), 141.5 (s, C-9), 122.6 (d, C-10), 82.0 (d, C-2), 72.0 (d, C-11), 71.5 (2 x d, C-7, C-8), 69.6 (d, C-3), 66.8 (s, C-12), 65.4 (t, C-15), 48.8 (s, C-13), 47.8 (s, C-6), 47.3 (s, C-5), 46.0 (t, C-4), 21.2 (q, acetyl CH_3_), 20.8 (q, C-16), 16.0 (q, C-14). HRMS (APCI^+^): m/z calcd for (**8**) [M + Na^+^]: 363.1414; found: 363.1411. NMR data for 7,8-dihydroxycalonectrin and 3-deacetyl-7,8-dihydroxycalonectrin were found in accordance with the ^1^H chemical shifts reported in literature [22].

## 5. Conclusions

This paper presents a reliable, mild, fast and tolerant method for the stereoselective reduction of the carbonyl group in deoxynivalenol and its acetylated derivatives. Although the method was optimized for deoxynivalenol, it is likely to be applicable to nivalenol, its acetylated derivatives and other type B trichothecenes in a similar way. The isolated yields of the method were satisfying and further improvements towards better yields of the presented method might be difficult to achieve due to the instability of several functional groups (epoxy, acetyl) with prolonged reaction time. Nevertheless, we expect the method to also be applicable on masked forms of mycotoxins thereby providing a valuable tool for synthetic conversion of different mycotoxin standards.

In addition to the reduction itself, four different calonectrin derivatives were synthesized and characterized using several NMR techniques. By using NOESY (Nuclear Overhauser Enhancement SpectroscopY) we were able to prove the stereochemistry of all reaction products. The value of the compounds itself is difficult to estimate—although they are supposed to act as precursors for the biosynthesis of DON, their natural occurrence is reported rarely in literature. Nonetheless, the protocol presented can be readily applied for the synthesis of this class of compounds and therefore opens the door for their use as reference material as well as for investigations on the biologic pathway towards type B trichothecenes.

## Supplementary Files

Supplementary File 1 Supplementary Information (PDF, 3090 KB)
