# Supplementary material for: Stereoselective Luche Reduction of Deoxynivalenol and Three of Its Acetylated Derivatives at C8
Source: Toxins (Basel). 2014 Jan 10;6(1):325–36. doi: 10.3390/toxins6010325 (PMC3920264; doi:10.3390/toxins6010325)
Supplement: Supplementary File 1 — Supplementary Information (PDF, 3090 KB) [file toxins-06-00325-s001.pdf]

Supplementary Information

1. NMR Spectra

1.1. Deoxynivalenol and Derivatives

Figure S1. ^{1}H (200 MHz, $CDCl_3$) of 3-ADON (**1**).

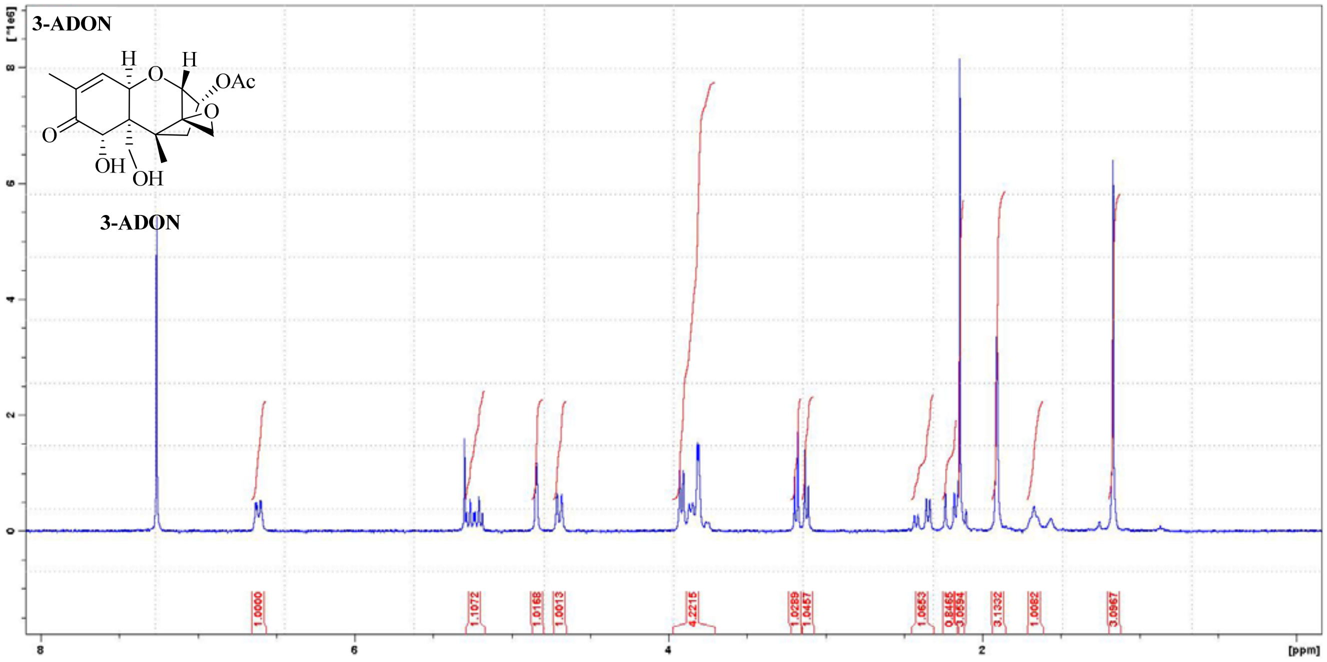

Figure S2. ^{1}H (200 MHz, $CDCl_3$) of 15-ADON (**3**).

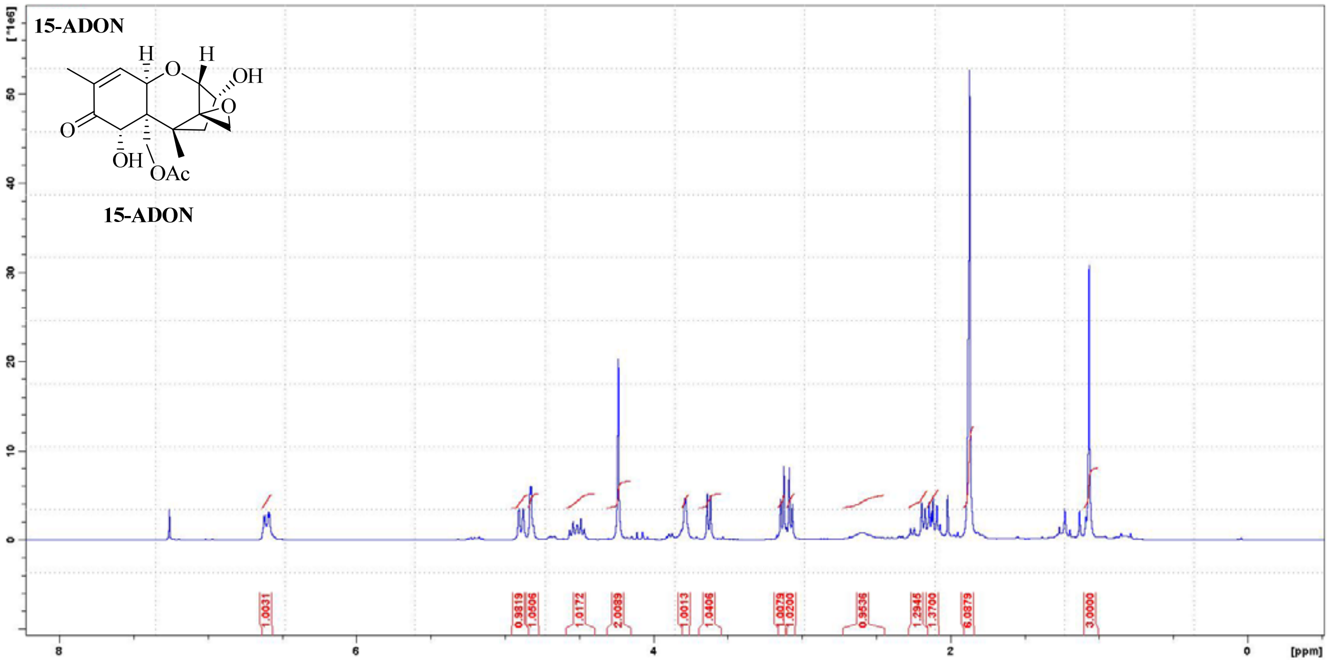

Figure S3. ^{13}C (50 MHz, $CDCl_3$) of 15-ADON (**3**).

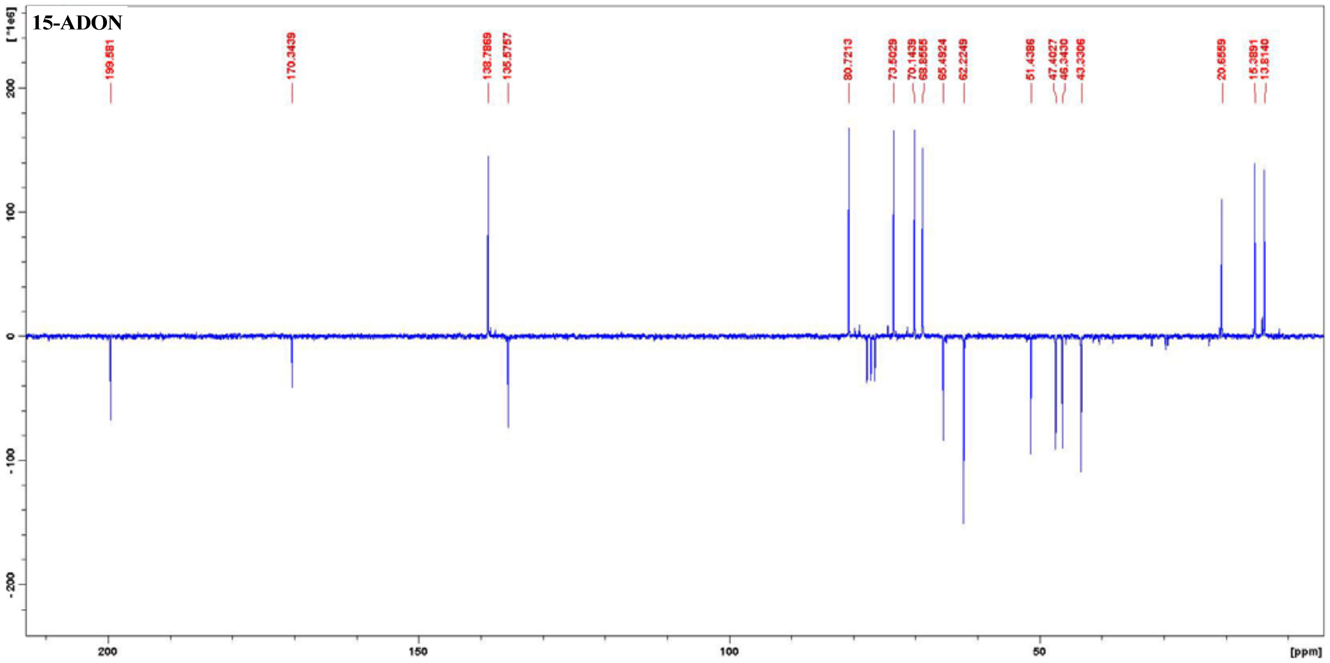

Figure S4. ^{1}H (200 MHz, $CDCl_3$) of 3,15-diADON (**4**).

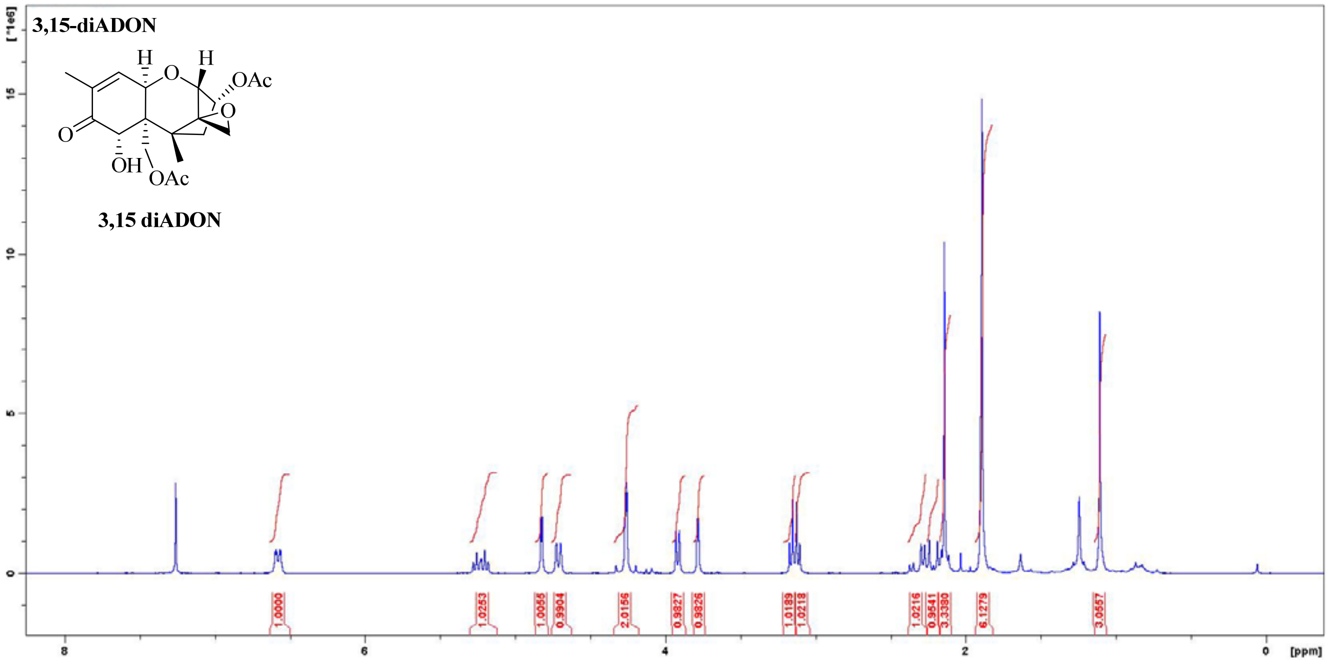

Figure S5. ^{13}C (50 MHz, $CDCl_3$) of 3,15-diADON (**4**).

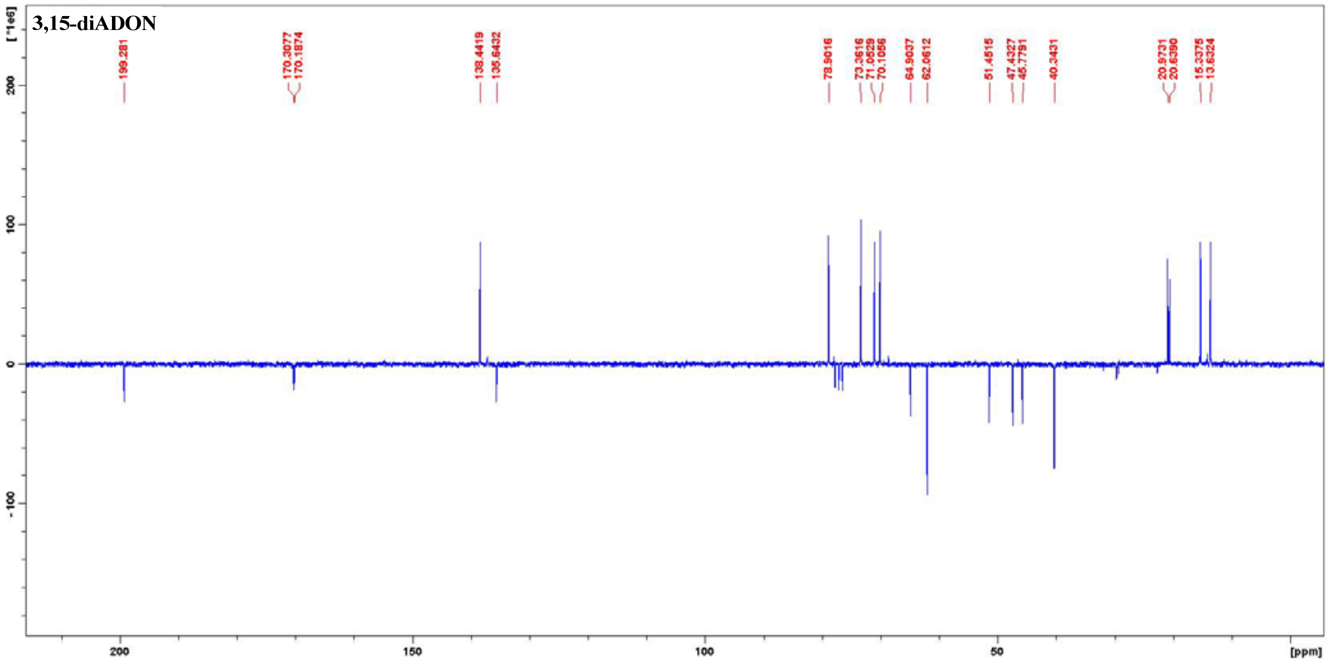

1.2. Screening Product

Figure S6. ^{1}H (200 MHz, $CDCl_3$) of (+)-*cis*-carveole (**11**).

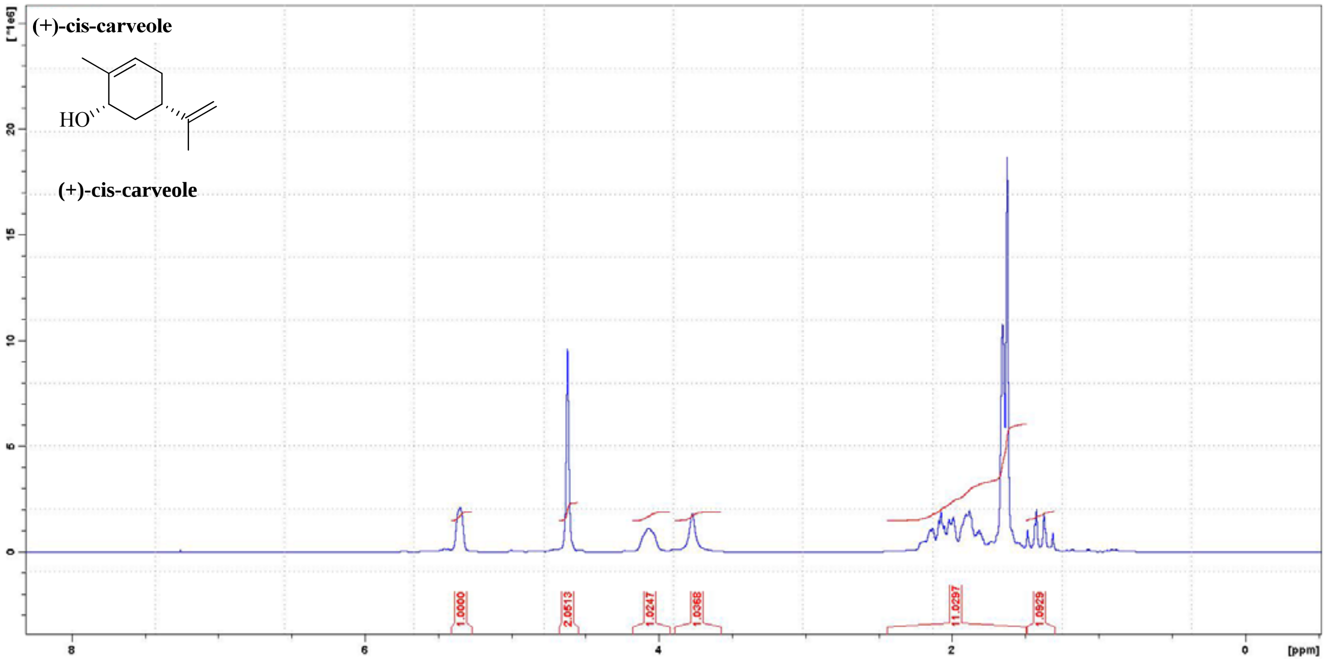

Figure S7. ^{13}C (50 MHz, $CDCl_3$) of (+)-*cis*-carveole (**11**).

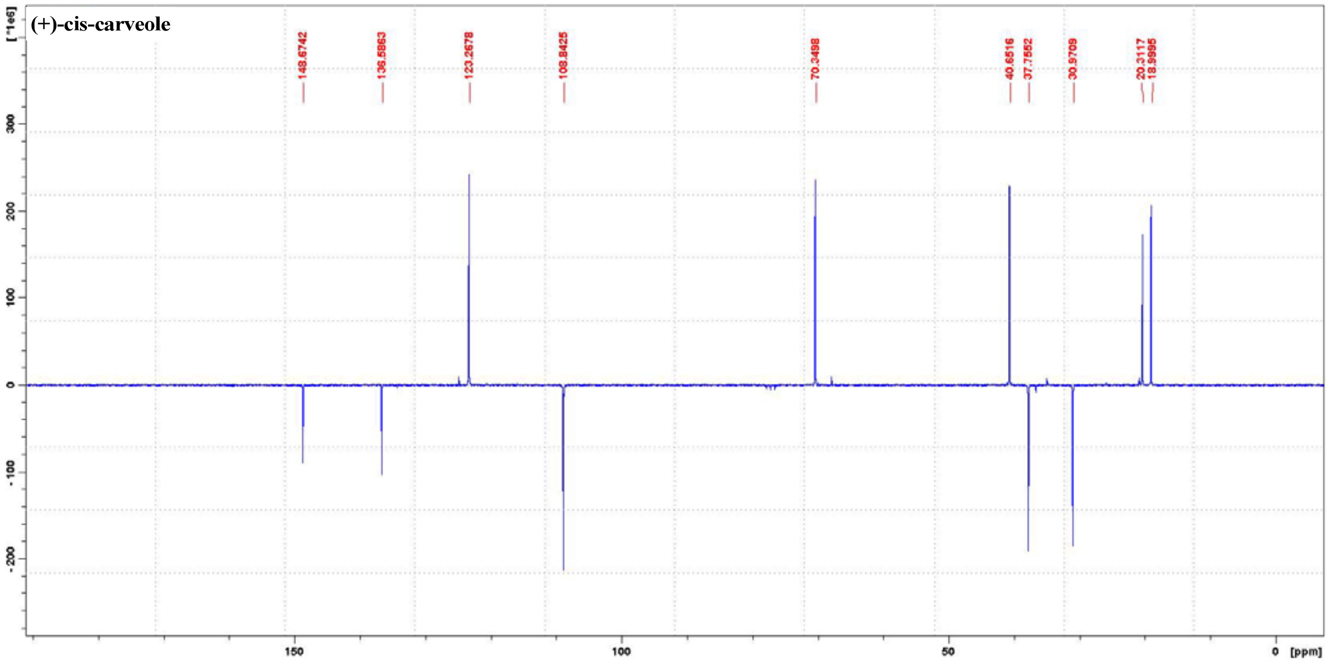

1.2. 7,8-Dihydroxycalonectrin and its Derivatives

Figure S8. ^{1}H (400 MHz, methanol-d_4) of 3,7,8,15-tetrahydroxyscirpene (**5**).

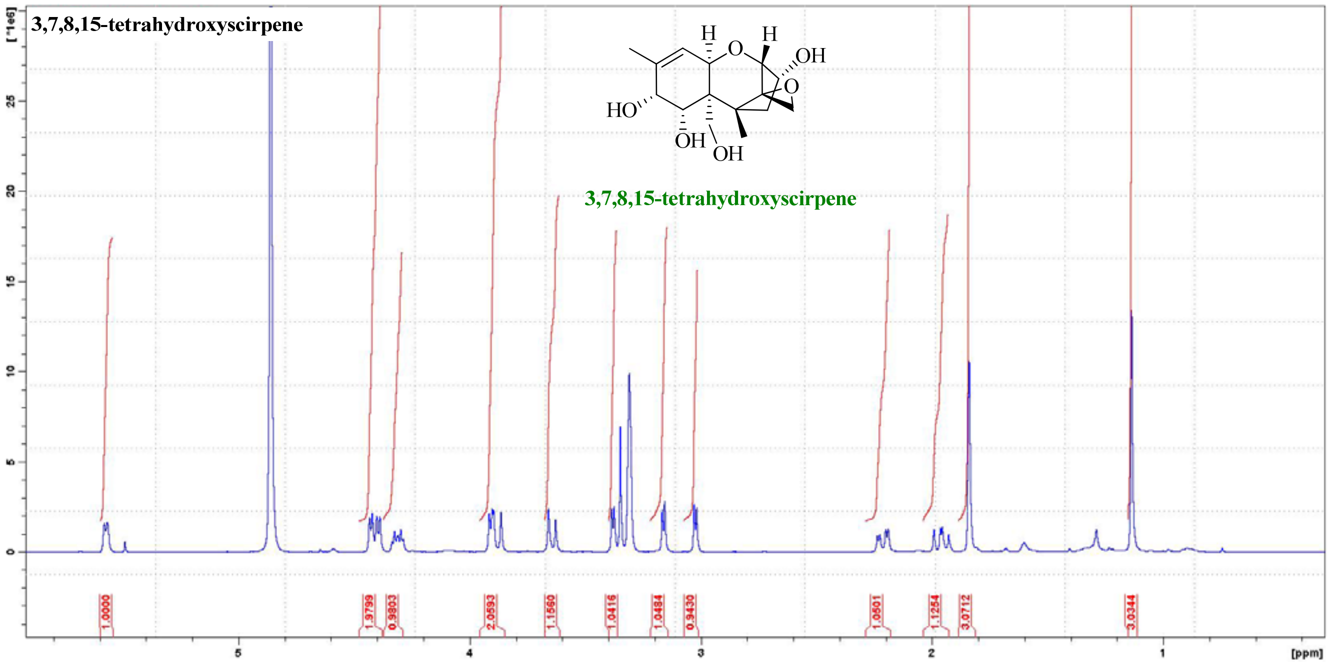

Figure S9. ^{13}C (100 MHz, methanol-d_4) of 3,7,8,15-tetrahydroxyscirpene (**5**).

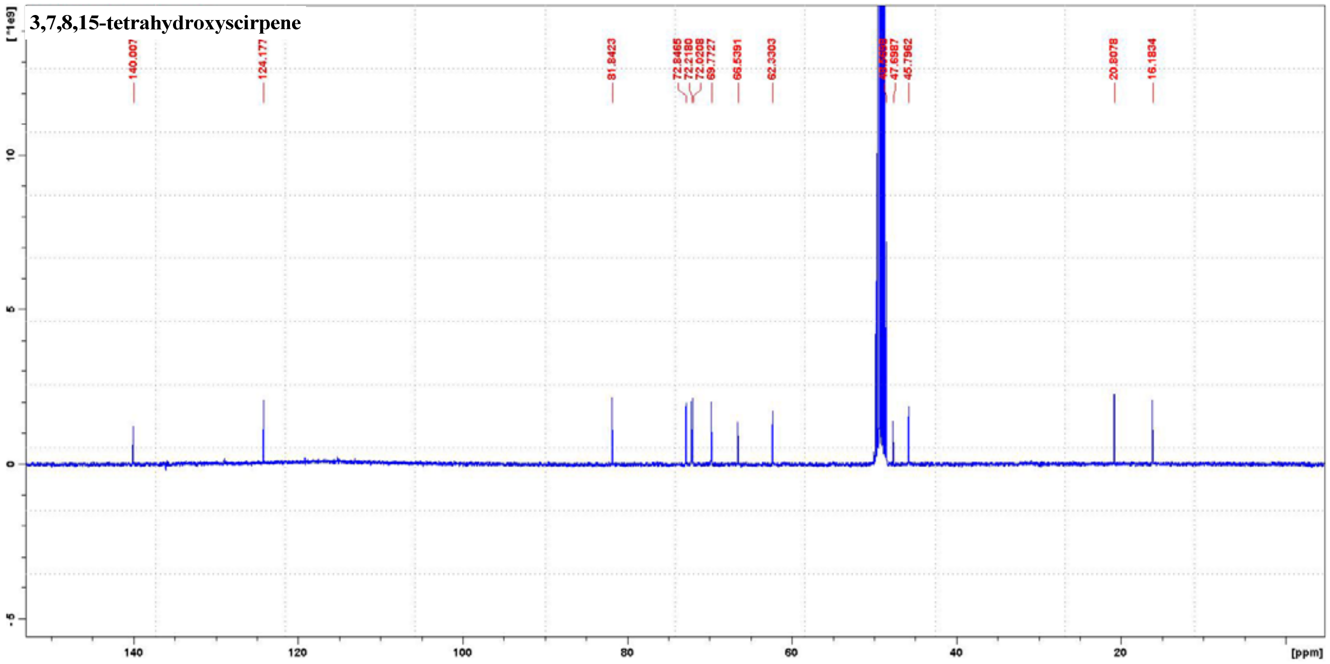

Figure S10. COSY (400 MHz, methanol-d_4) of 3,7,8,15-tetrahydroxyscirpene (**5**).

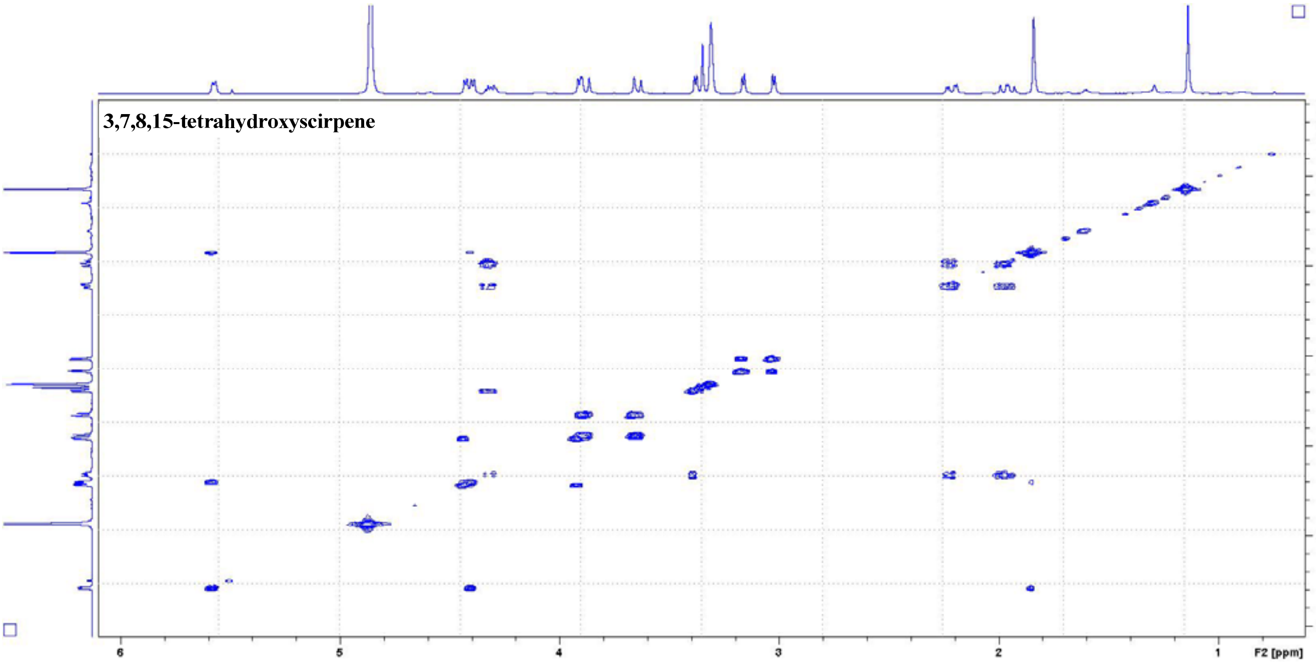

Figure S11. HSQC (400 MHz, methanol-d_4) of 3,7,8,15-tetrahydroxyscirpene (**5**).

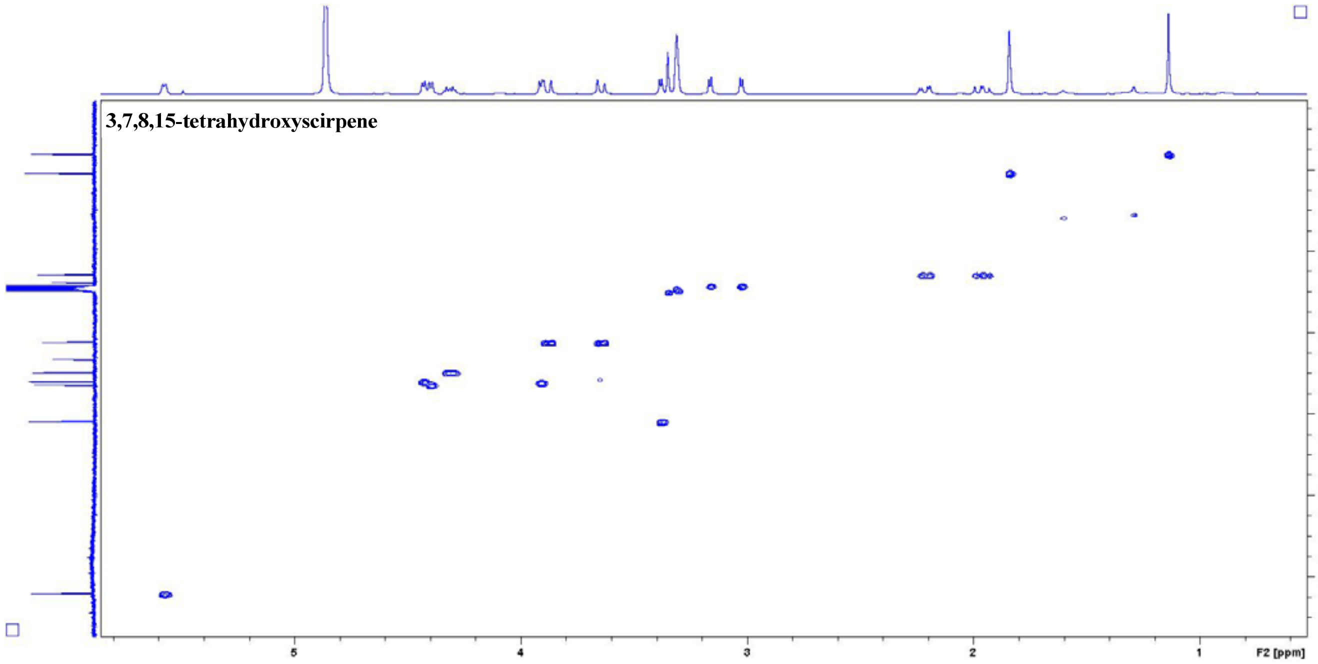

Figure S12. HMBC (400 MHz, methanol-d_4) of 3,7,8,15-tetrahydroxyscirpene (**5**).

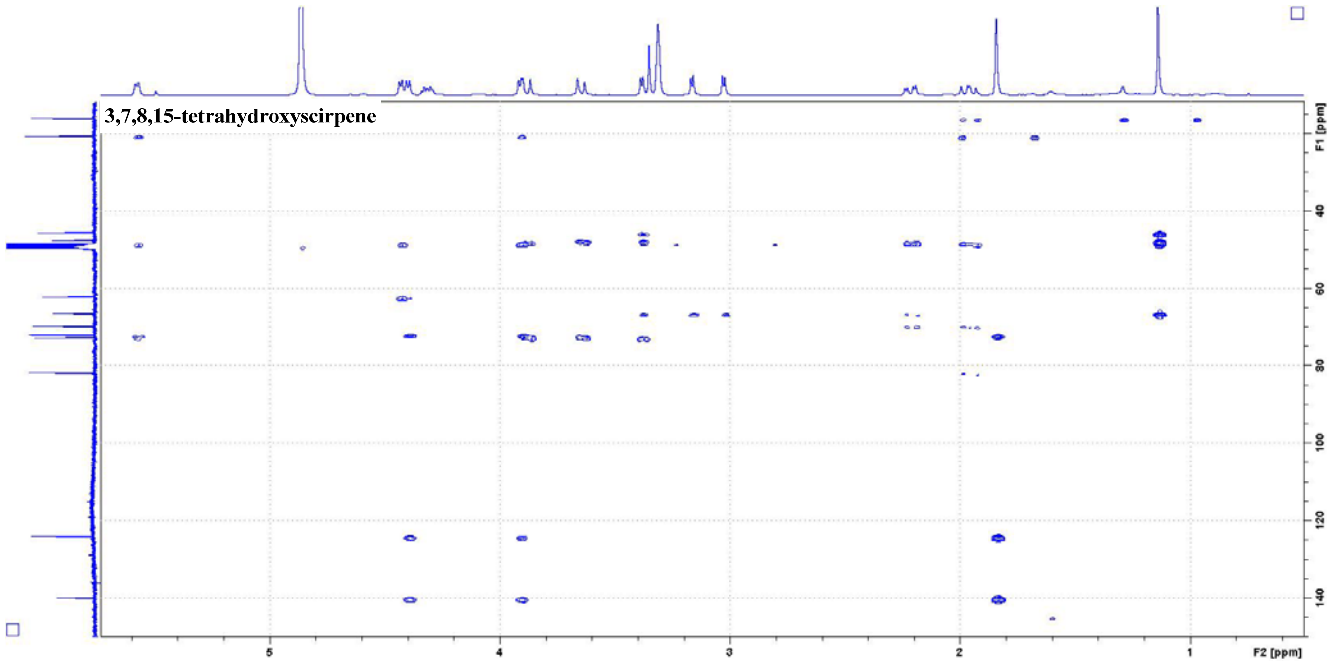

Figure S13. ^{1}H (400 MHz, methanol-d_4) of 15-deacety-7,8-dihydroxylcalonectrin (**7**).

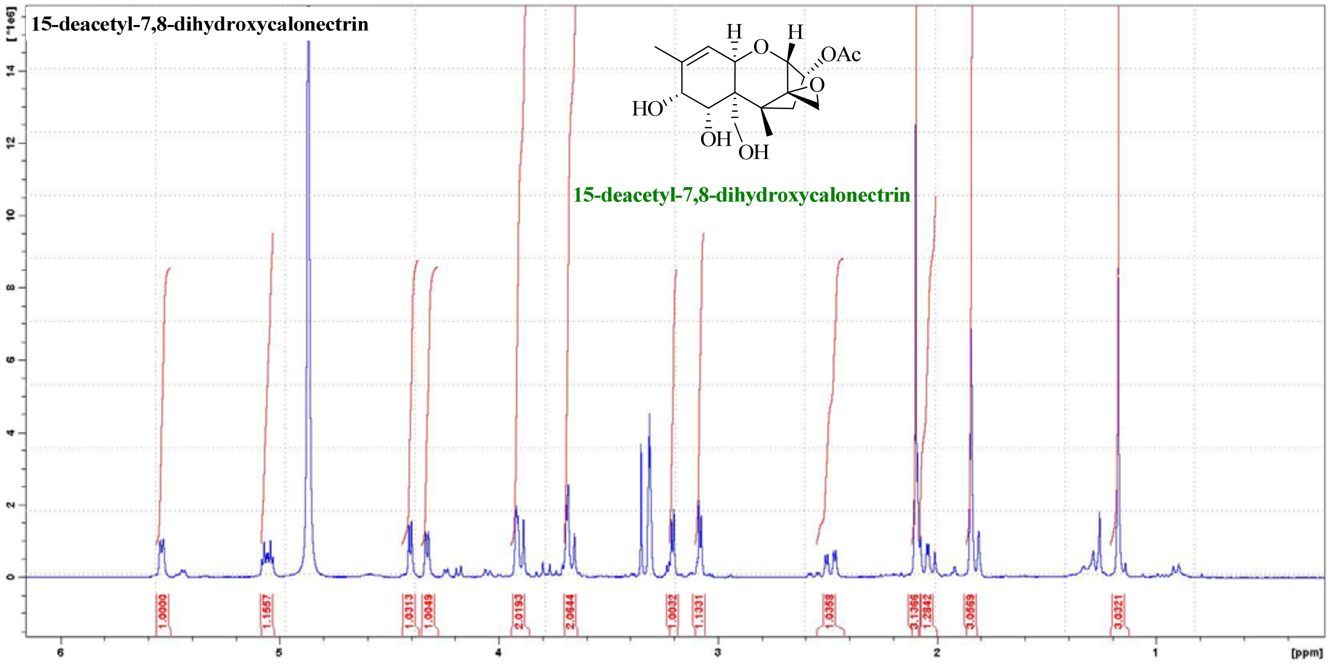

Figure S14. ^{13}C (100 MHz, methanol-d_4) of 15-deacetyl-7,8-dihydroxycalonectrin (**7**).

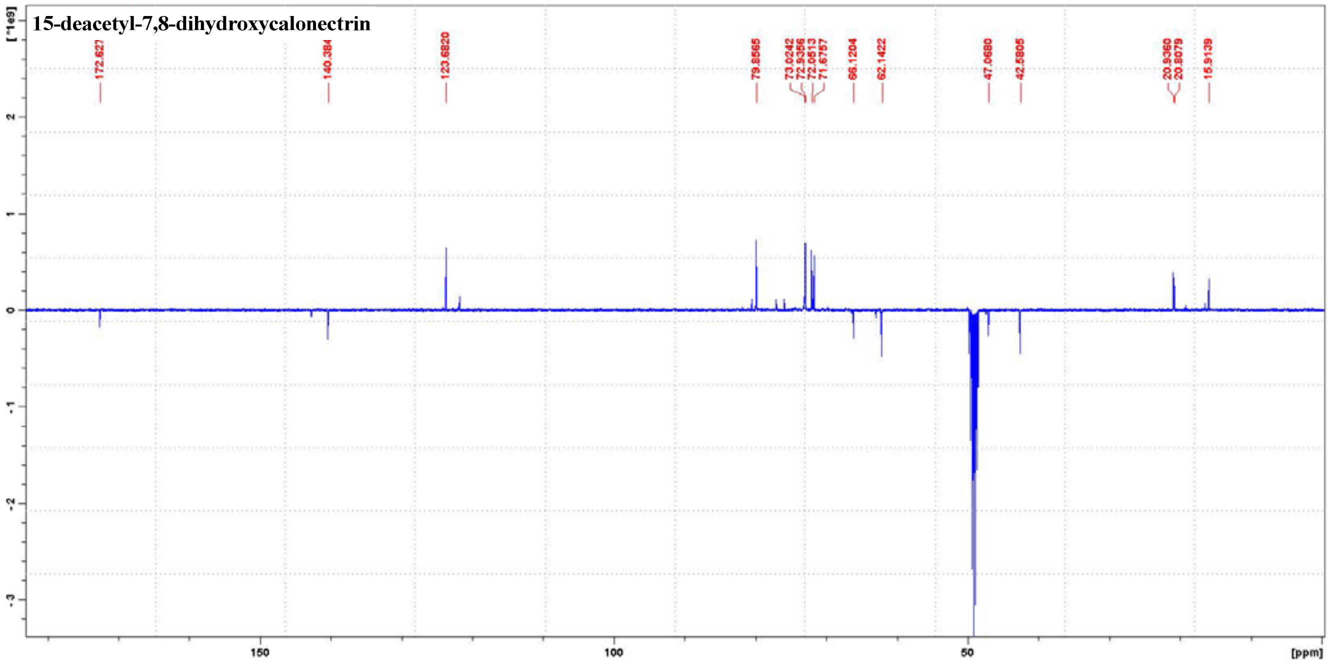

Figure S15. COSY (400 MHz, methanol-d_4) of 15-deacetyl-7,8-dihydroxycalonectrin (**7**).

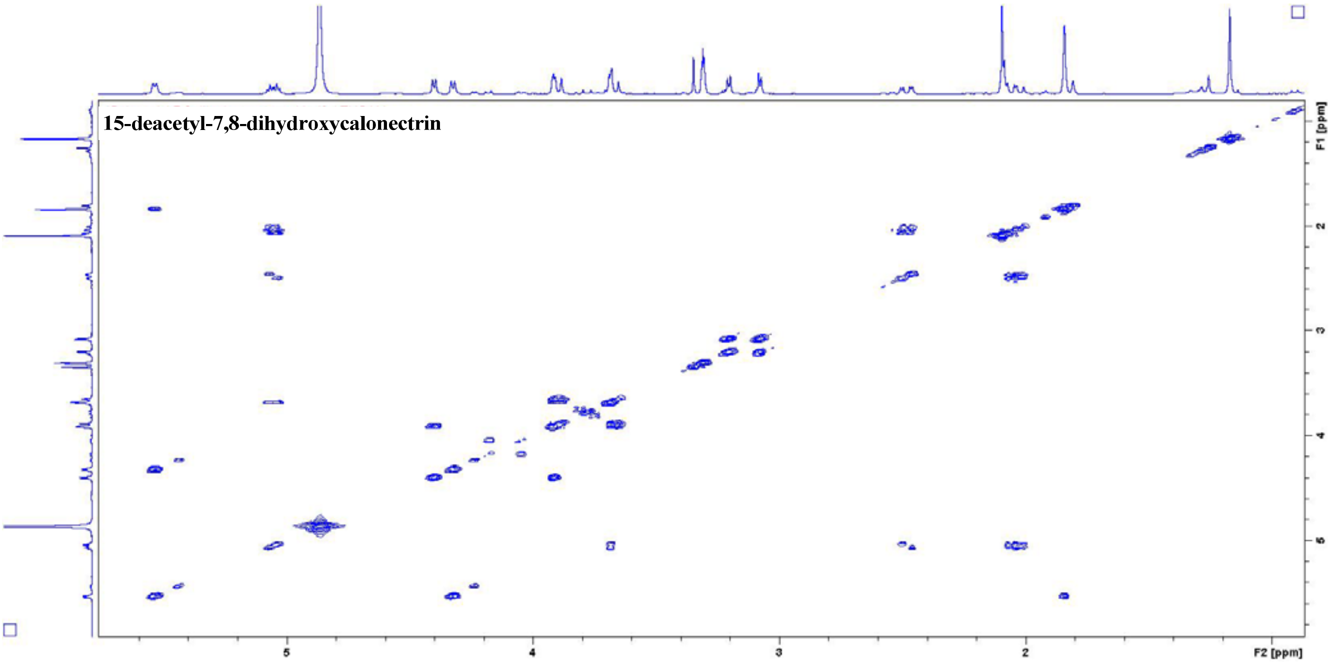

Figure S16. HSQC (400 MHz, methanol-d_4) of 15-deacetyl-7,8-dihydroxycalonectrin (**7**).

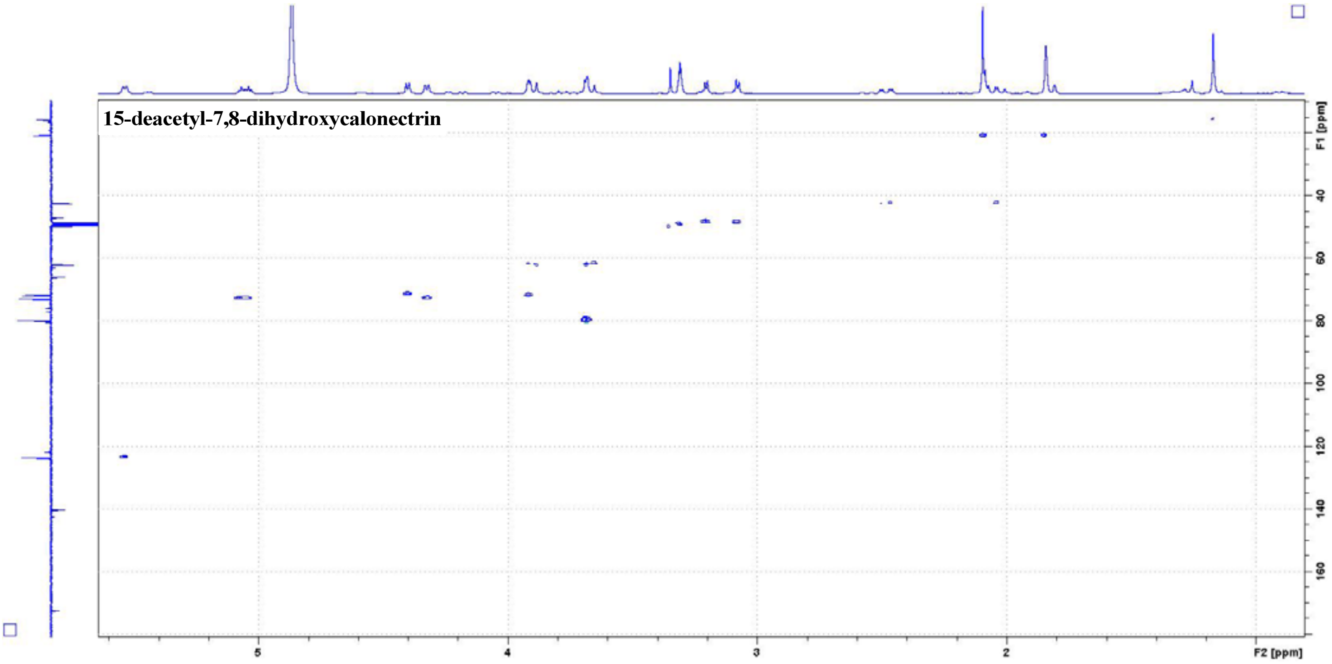

Figure S17. HMBC (400 MHz, methanol-d_4) of 15-deacetyl-7,8-dihydroxycalonectrin (**7**).

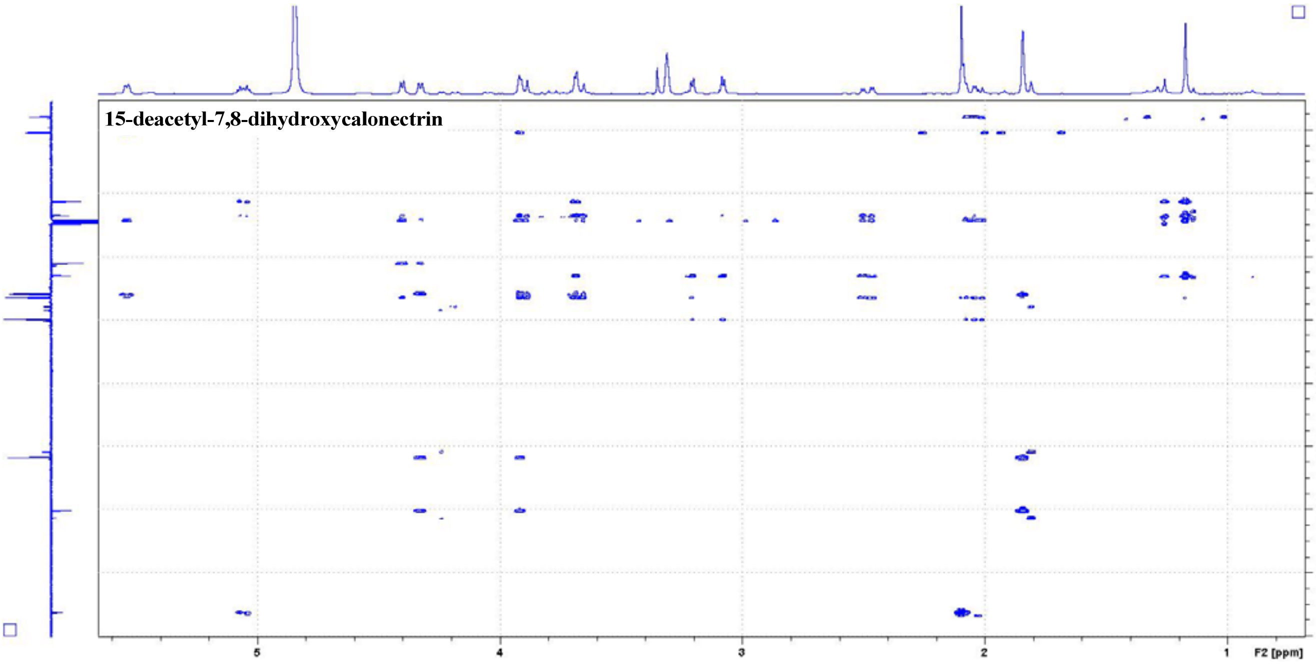

Figure S18. ^{1}H (400 MHz, methanol-d_4) of 3-deacetyl-7,8-dihydroxycalonectrin (**8**).

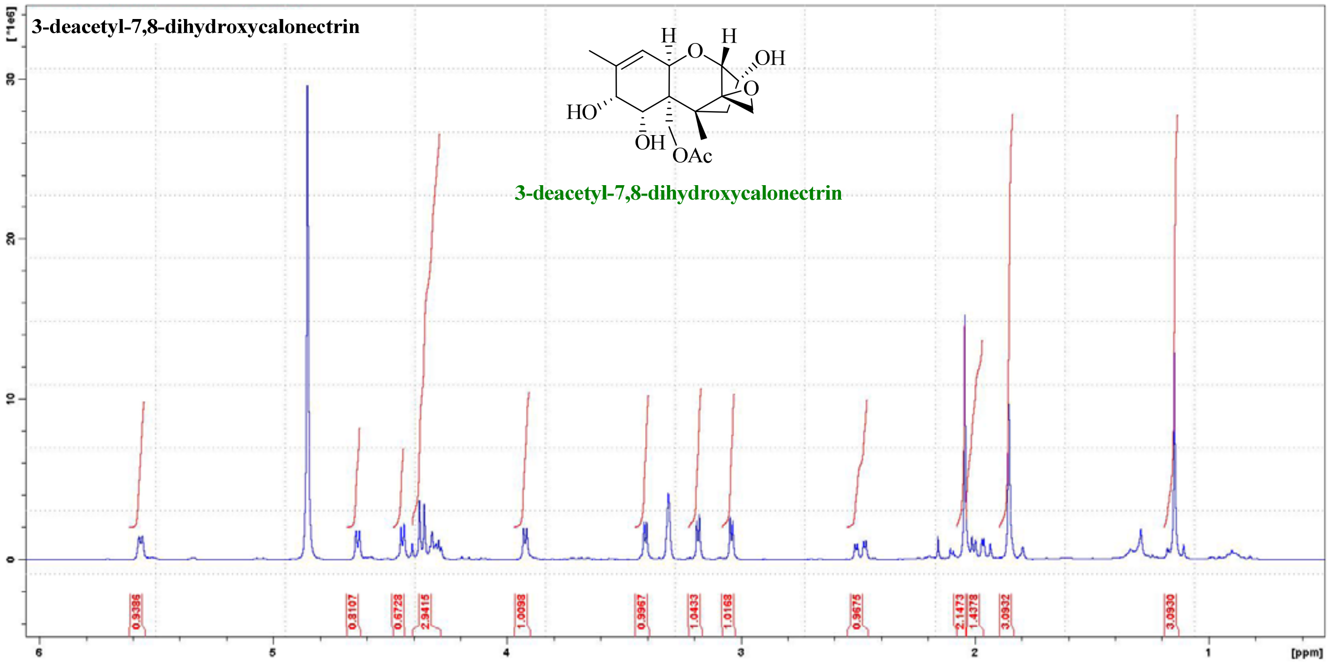

Figure S19. ^{13}C (100 MHz, methanol-d_4) of 3-deacetyl-7,8-dihydroxycalonectrin (**8**).

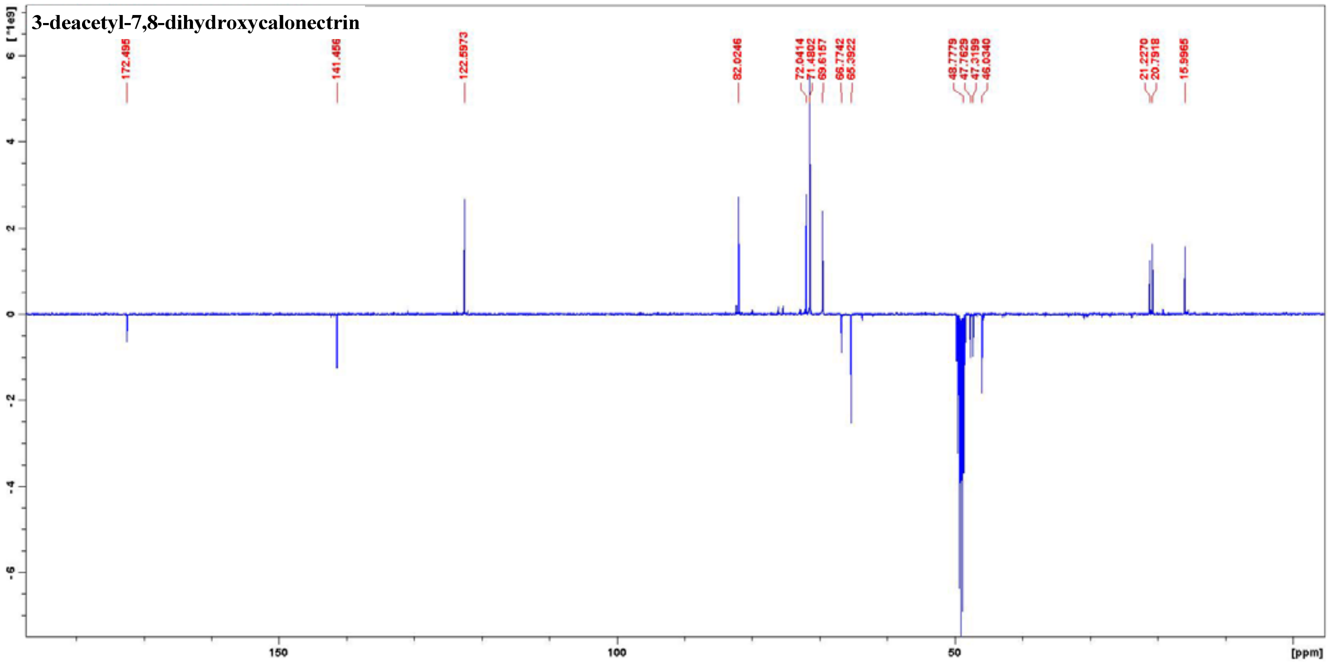

Figure S20. COSY (400 MHz, methanol-d_4) of 3-deacetyl-7,8-dihydroxycalonectrin (**8**).

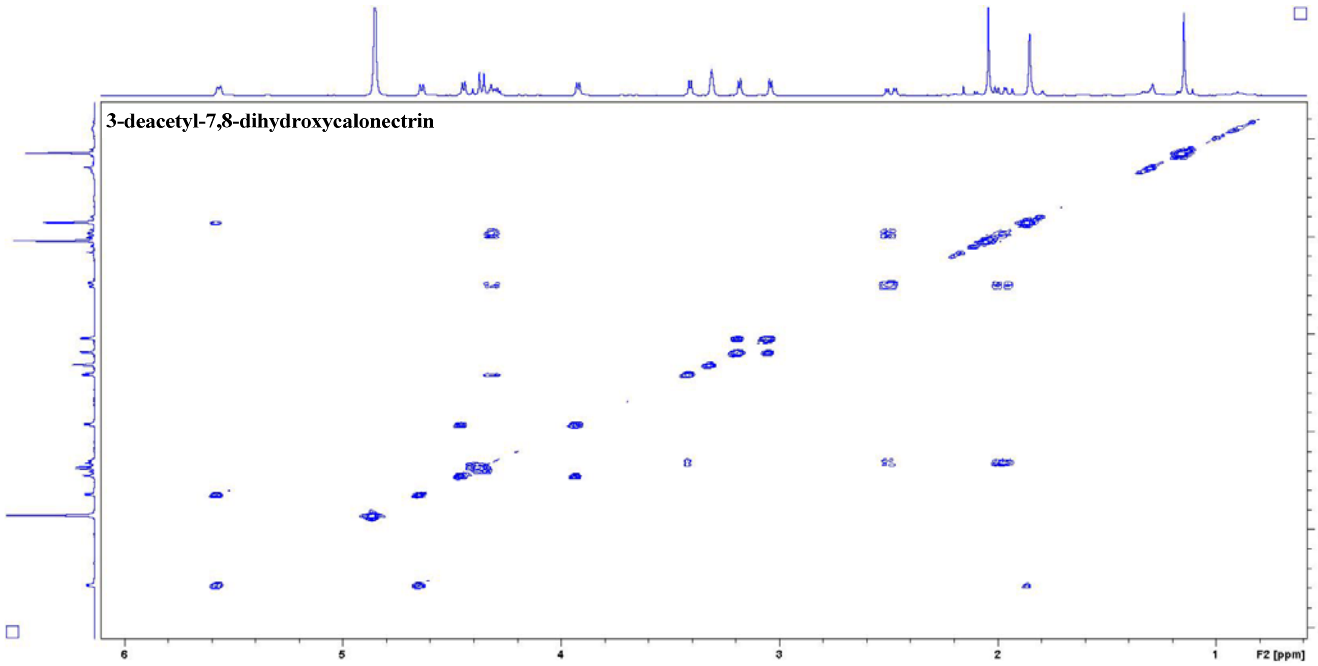

Figure S21. HSQC (400 MHz, methanol-d_4) of 3-deacetyl-7,8-dihydroxycalonectrin (**8**).

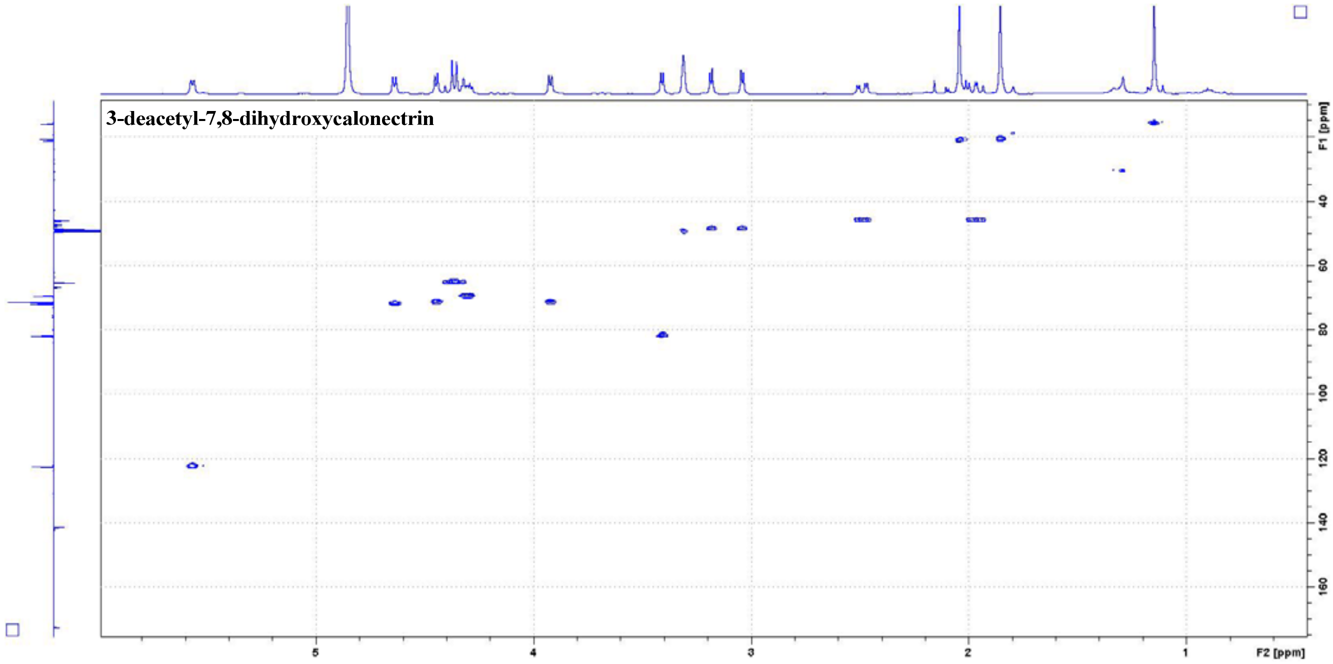

Figure S22. HMBC (400 MHz, methanol-d_4) of 3-deacetyl-7,8-dihydroxycalonectrin (**8**).

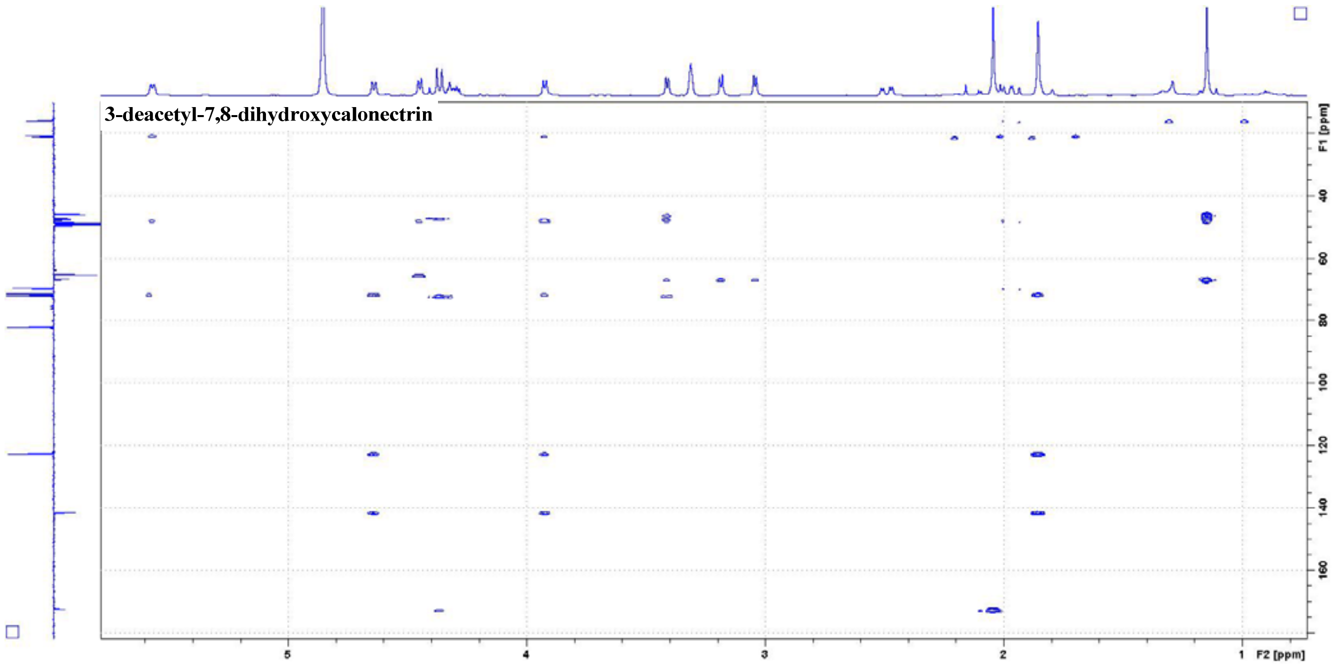

Figure S23. ^{1}H (200 MHz, methanol-d_4) of 7,8-dihydroxycalonectrin (**9**).

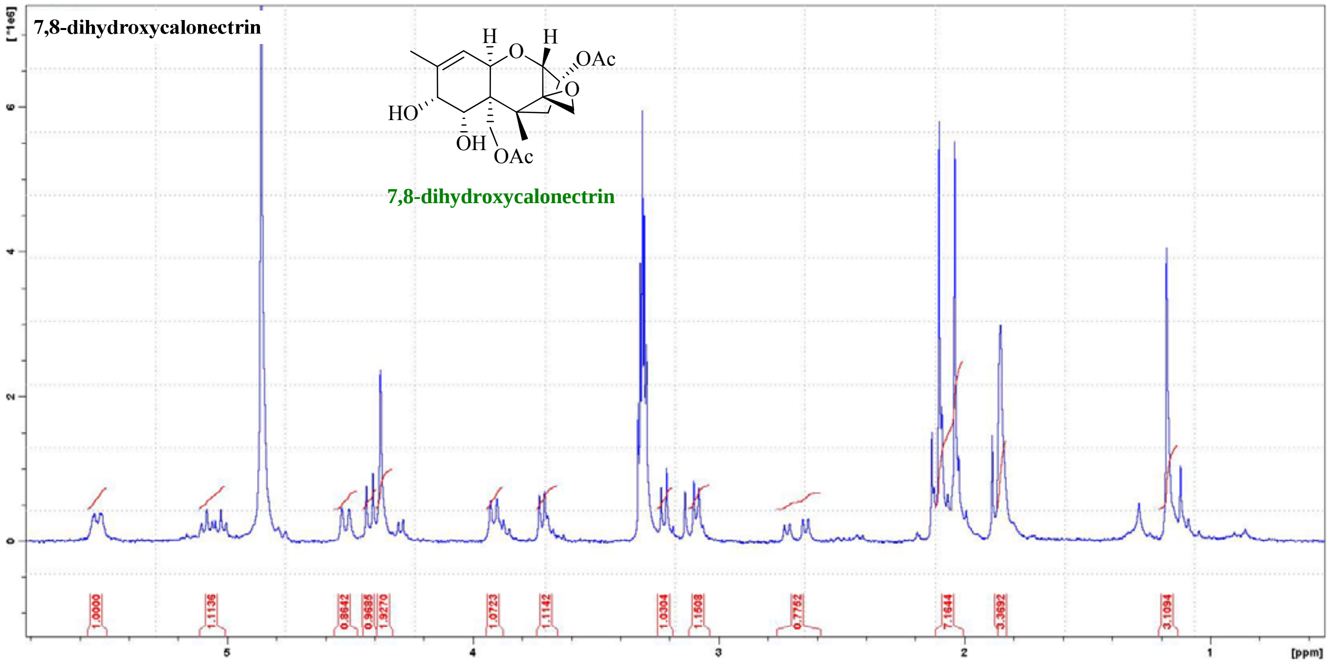

Figure S24. ^{13}C (50 MHz, methanol-d_4) of 7,8-dihydroxycalonectrin (**9**).

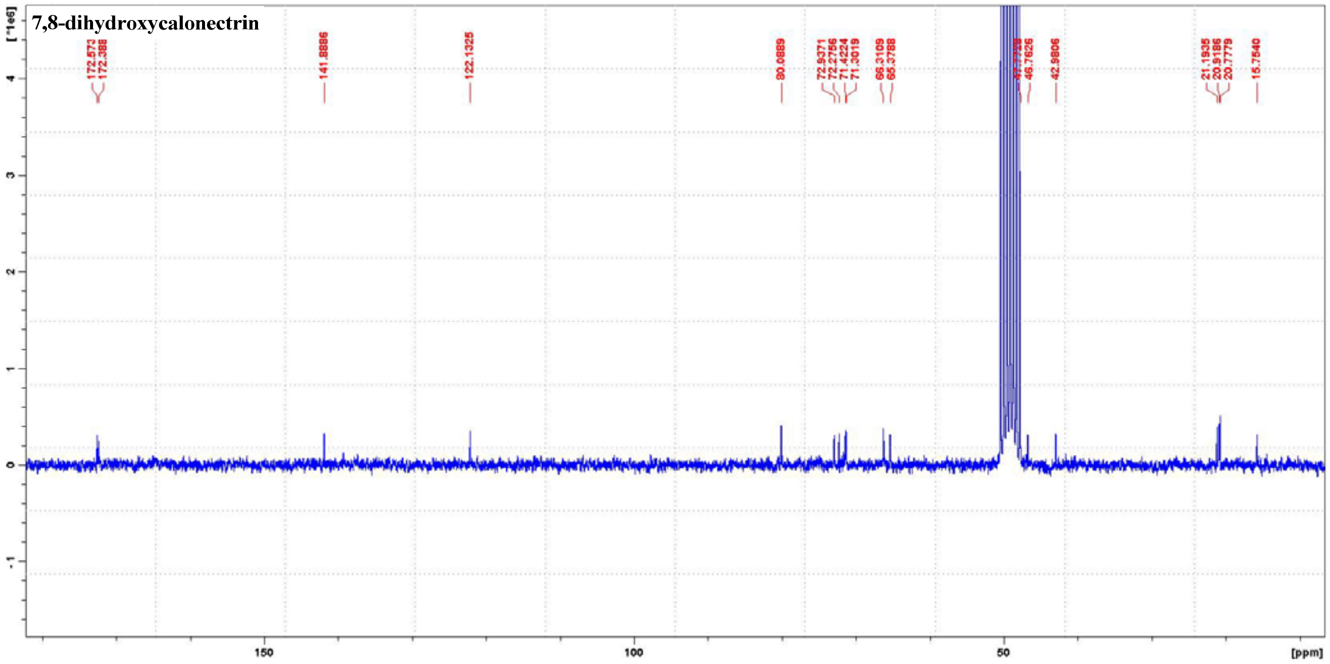

2. CD Spectra

Figure S25. CD spectra of Deoxynivalenol (**2**), 1 mmol in H_2O.

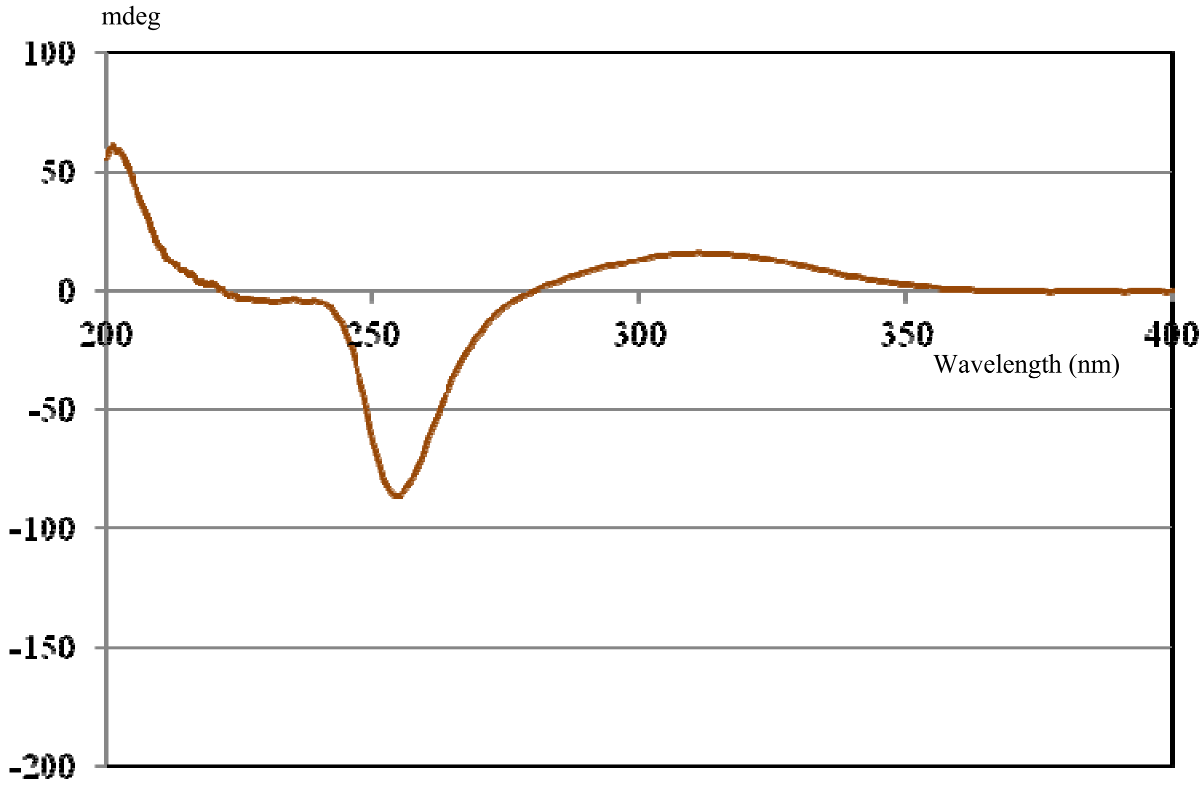

Figure S26. CD spectra of 3,7,8,15-Tetrahydroxyscirpene (**5**), 1 mmol in H_2O.

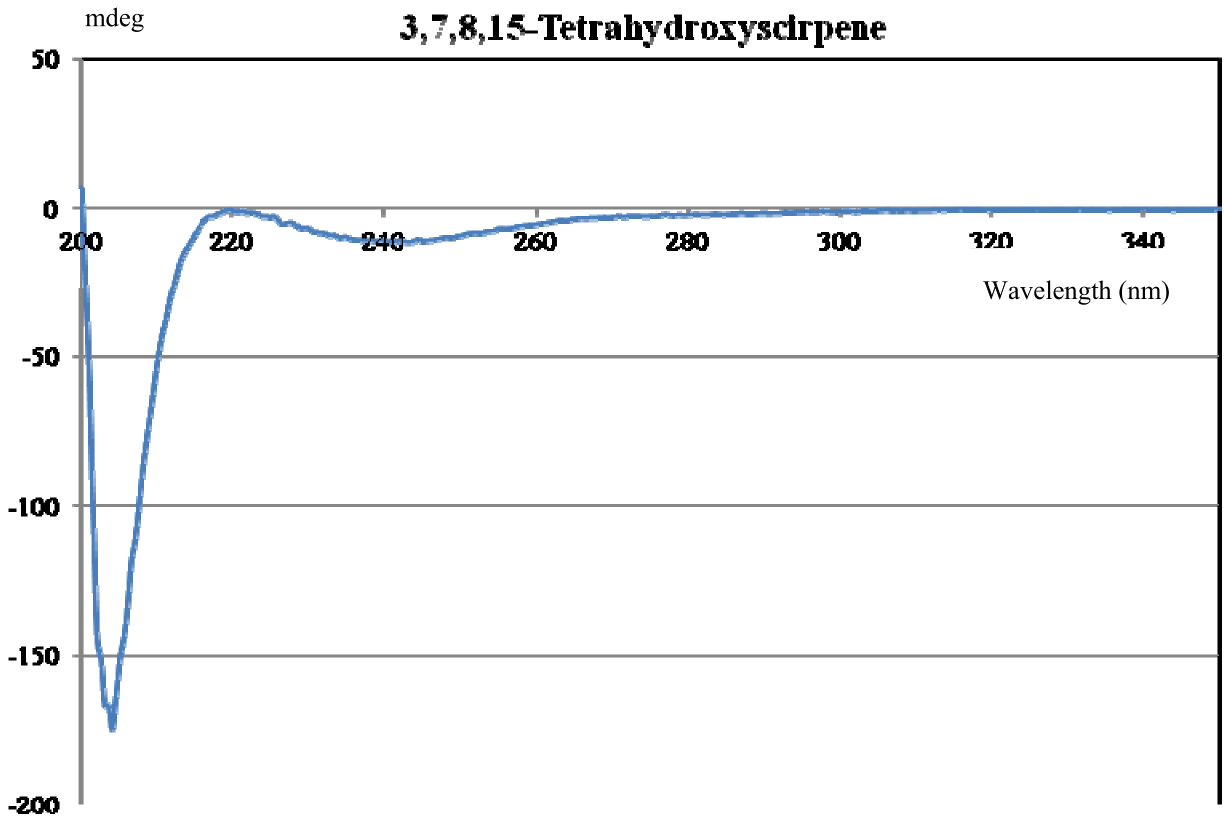

Figure S27. CD spectra of 15-Deacetyl-7,8-dihydroxycalonectrin (**7**), 1 mmol in H_2O.

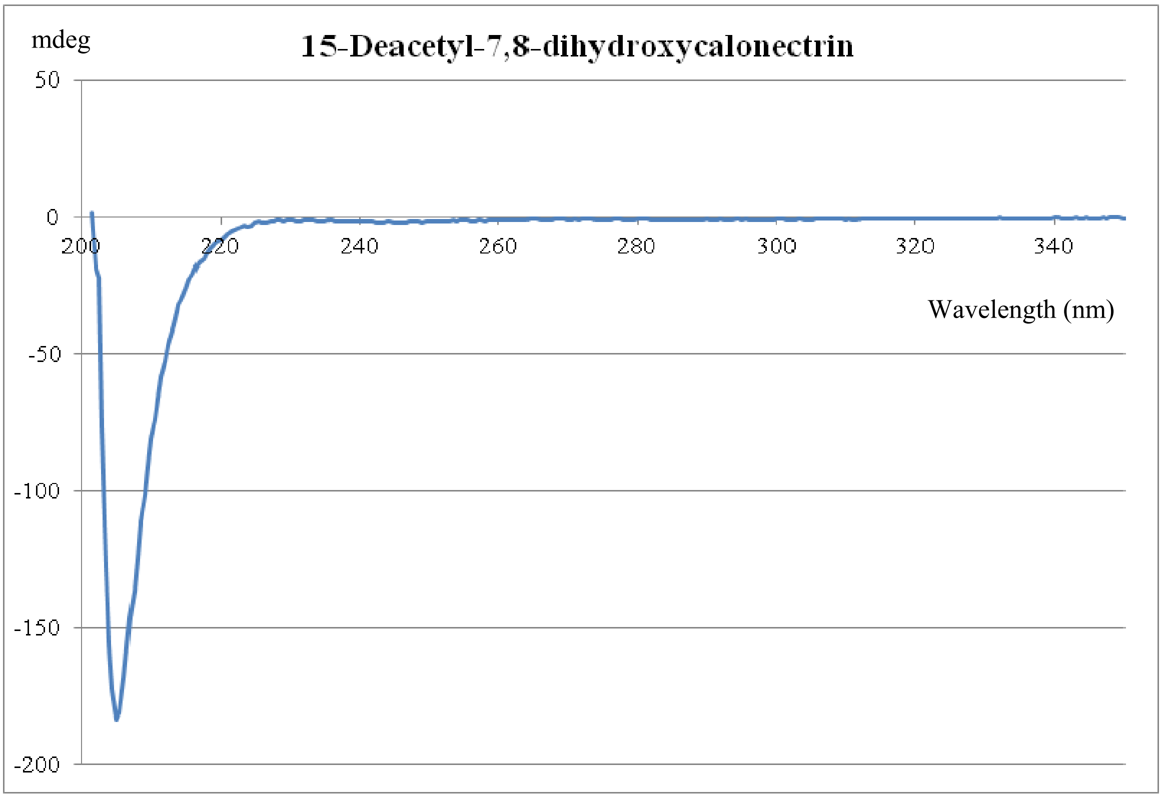

Figure S28. CD spectra of 3-Deacetyl-7,8-dihydroxycalonectrin (**8**), 1 mmol in H_2O.

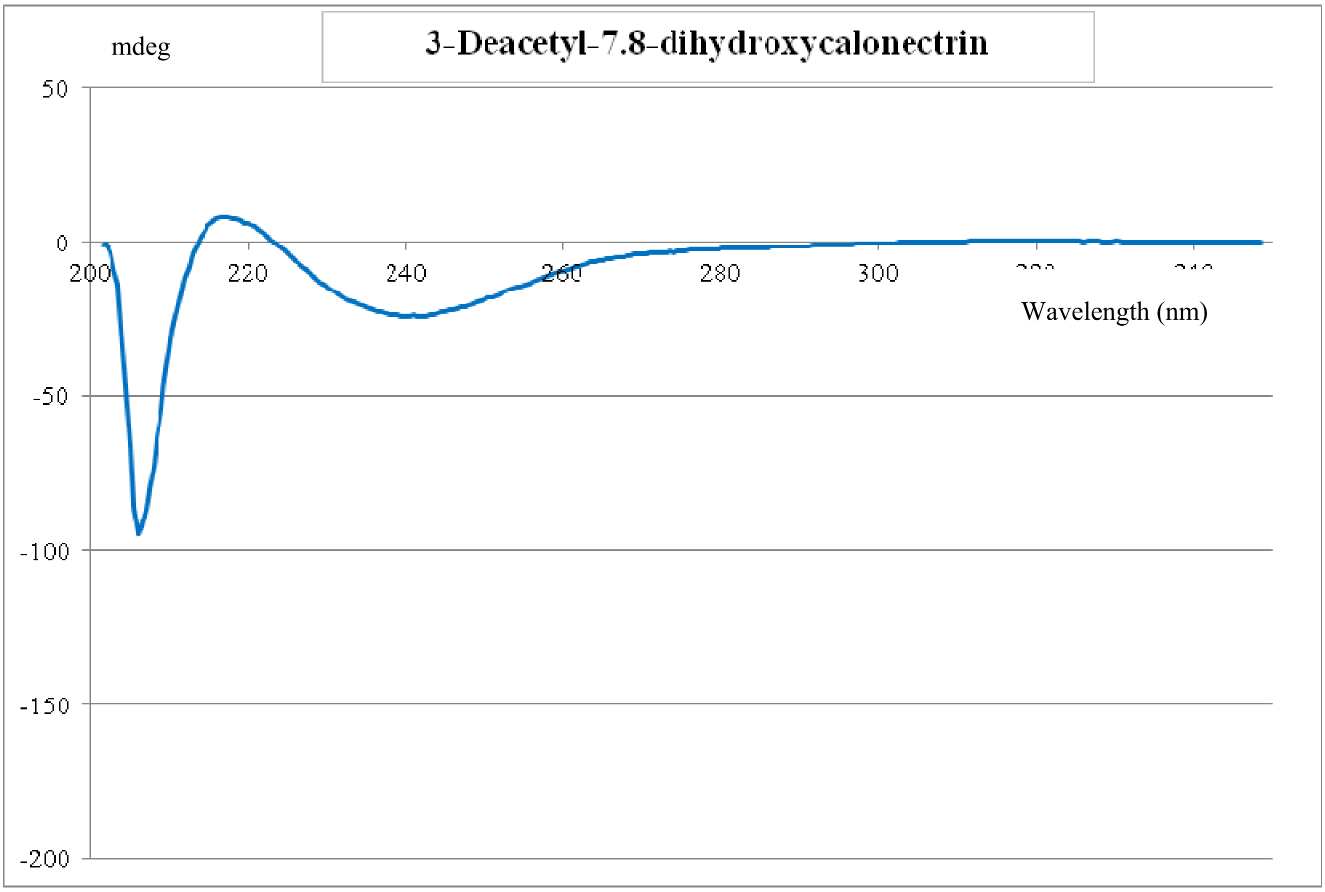

Figure S29. CD spectra of 7,8-dihydroxycalonectrin (**9**), 1 mmol in H_2O.

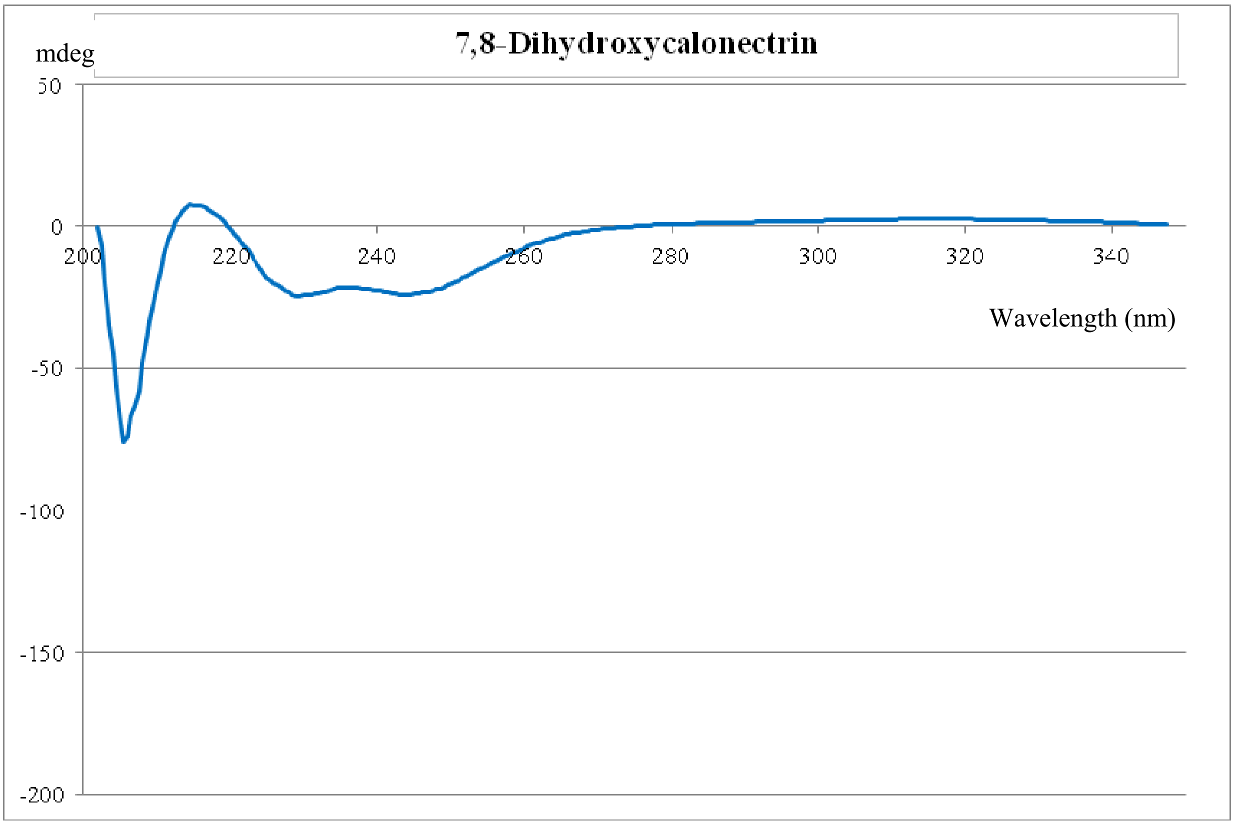

3. Quantum Chemical Calculations

On the basis of the observed NOESY correlations a reasonable starting geometry of **5** was optimized (geometry optimization to energy minimum as well as subsequent frequency analysis) applying DFT calculations [1,2] at the level 6-311++G(d,p) [3], using the B3LYP hybrid functional [4], as implemented in the Gaussian 09 (Revision A.1) program package [5]. Data analysis was done using GaussView 5 (Gaussian, Inc., Wallingford, CT, USA). The obtained geometry is shown in Figure S25 including selected NOESY correlations as indicated by dashed bonds. The determined H,H-distances for the selected NOESY correlations are summarized in Table S1.

Figure S30. Optimized geometry of **5** (selected NOESY correlations are indicated by dashed bonds).

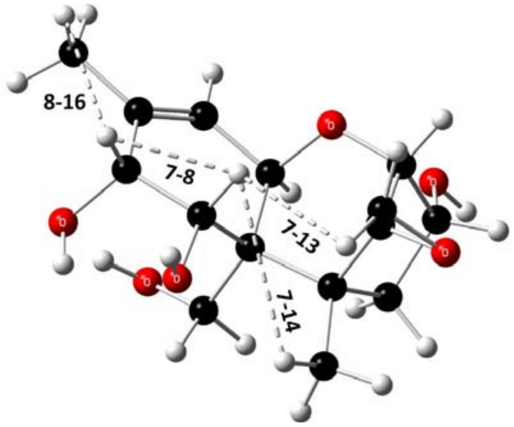

Table S1. Determined H,H-distances (r_{HH}, in Å) for selected NOESY correlations on the basis of the optimized geometry of **5** in gas phase.

**NOESY correlation**	**r_{HH} (5)**
H7–H8	2.32
H7–H13	2.69
H7–H14	3.29
H8–H16	2.57

Table S2. Optimized geometry for 5 in gas phase (DFT, 6-311++G(d,p), B3LYP).

**Nr.**	**Atom**	***Z*-matrix**						**Cartesian coordinates**		
		**NA**	**NB**	**NC**	**Bond**	**Angle**	**Dihedral**	***X***	***Y***	***Z***
**1**	C							2.60957	0.93565	−0.77360
**2**	C	1			1.50487			2.48377	−0.56222	−0.70144
**3**	C	2	1		1.54324	114.02722		1.03425	−1.06357	−0.53077
**4**	C	3	2	1	1.55203	114.53607	−44.52659	0.20196	−0.28298	0.52128
**5**	C	4	3	2	1.60130	113.24874	174.16170	−1.33530	−0.72882	0.56839
**6**	C	5	4	3	1.57383	110.14051	−164.74757	−2.18572	0.33978	1.35058
**7**	C	6	5	4	1.55635	107.04738	88.94198	−2.66054	1.39543	0.31021
**8**	C	7	6	5	1.54093	103.97291	−5.77396	−1.94792	0.98136	−0.99179
**9**	C	4	3	2	1.56192	105.22625	56.89452	0.24761	1.20752	0.05664
**10**	C	1	2	3	1.33142	119.91003	14.69751	1.58895	1.70621	−0.40313
**11**	C	8	7	6	1.51708	100.51706	33.90298	−1.93356	−0.52720	−0.83189
**12**	C	11	8	7	1.46136	124.83681	147.80617	−2.01451	−1.48422	−1.93331
**13**	C	1	10	9	1.50389	123.57680	179.40739	3.93320	1.47601	−1.24016
**14**	C	4	3	2	1.56325	112.18598	−64.44281	0.77327	−0.44855	1.96694
**15**	C	5	4	3	1.53483	114.75570	70.14387	−1.58087	−2.12947	1.14593
**16**	O	8	7	6	1.42110	113.99811	−82.49657	−0.60939	1.44817	−1.09167
**17**	O	2	1	10	1.44275	108.20024	−109.75966	3.33159	−1.03473	0.36602
**18**	O	3	2	1	1.44558	107.22092	−168.71583	1.10328	−2.47155	−0.21057
**19**	O	7	6	5	1.42388	115.36349	−126.84526	−2.35864	2.74890	0.63334
**20**	O	12	11	8	1.43810	59.64435	−101.13525	−3.17643	−1.19131	−1.13815
**21**	O	14	4	3	1.41596	116.73478	80.54735	1.88059	0.36402	2.31120
**22**	H	2	1	10	1.10182	108.76104	133.84972	2.86358	−0.99540	−1.64064
**23**	H	3	2	1	1.09226	106.48716	74.46622	0.54691	−0.93878	−1.50028
**24**	H	6	5	4	1.08978	113.21654	−32.58852	−1.63123	0.82684	2.15241
**25**	H	7	6	5	1.09526	109.69558	108.54902	−3.73668	1.28615	0.13826
**26**	H	8	7	6	1.09006	113.20362	156.08476	−2.45767	1.33008	−1.89000
**27**	H	9	4	3	1.09252	108.88526	−165.60008	−0.09428	1.84402	0.87615
**28**	H	10	1	13	1.08596	119.89627	2.46129	1.67549	2.78683	−0.46710
**29**	H	12	11	8	1.08745	119.82552	156.47846	−1.62569	−2.49154	−1.80424
**30**	H	12	11	8	1.08785	119.70215	2.34656	−2.05308	−1.12364	−2.95894

Table S2. *Cont.*

Nr.	Atom	*Z*-matrix						Cartesian coordinates		
		NA	NB	NC	Bond	Angle	Dihedral	*X*	*Y*	*Z*
**31**	H	13	1	10	1.09622	110.94873	−121.04216	4.16116	1.13166	−2.25562
**32**	H	13	1	10	1.09133	111.27095	−0.44044	3.93752	2.56734	−1.24013
**33**	H	13	1	10	1.09441	110.76251	120.32235	4.74454	1.12577	−0.59457
**34**	H	14	4	3	1.08931	108.76775	−161.13599	−0.00270	−0.18121	2.68318
**35**	H	14	4	3	1.09518	108.31045	−45.34996	1.00220	−1.50747	2.12725
**36**	H	15	5	4	1.09220	110.98074	67.36836	−1.33830	−2.16162	2.21037
**37**	H	15	5	4	1.09201	108.95594	−174.23921	−2.63922	−2.37652	1.03947
**38**	H	15	5	4	1.08777	112.48858	−53.26508	−0.98718	−2.89407	0.64977
**39**	H	17	2	1	0.96784	106.25624	169.55318	3.10603	−1.96649	0.49891
**40**	H	18	3	2	0.96289	108.45933	−80.84470	1.22914	−2.96434	−1.02818
**41**	H	19	7	6	0.96198	108.59281	−75.02484	−2.95981	3.04043	1.32545
**42**	H	21	14	4	0.96949	107.35043	−71.65088	2.64948	0.02483	1.82781
**43**	H	6	5	4	1.09230	108.68967	−151.71110	−3.04324	−0.16055	1.80605

References

1. Kohn, W.; Sham, L.J. Self-consistent equations including exchange and correlation effects. *Phys. Rev.* **1965**, *140*, A1133–A1138.
2. Parr, R.G.; Yang, W. *Density Functional Theory of Atoms and Molecules*. Oxford University Press: London, UK, 1989.
3. Krishnan, R.; Binkley, J.S.; Seeger, R.; Pople, J.A. Self consistent molecular orbital methods. XX. A basis set for correlated wave functions. *J. Chem. Phys.* **1980**, *72*, 650–654.
4. Becke, A.D. Density-functional thermochemistry. III. The role of exact exchange. *J. Chem. Phys.* **1993**, *98*, 5648–5652.
5. Frisch, M.J.; Trucks, G.W.; Schlegel, H.B.; Scuseria, G.E.; Robb, M.A.; Cheeseman, J.R.; Scalmani, G.; Barone, V.; Mennucci, B.; Petersson, G.A.; *et al. Gaussian 09, Revision A.1*. Gaussian Inc: Wallingford, CT, USA, 2009.

© 2014 by the authors; licensee MDPI, Basel, Switzerland. This article is an open access article distributed under the terms and conditions of the Creative Commons Attribution license (http://creativecommons.org/licenses/by/3.0/).
